# Genomic Insights into a New *Citrobacter koseri* Strain Revealed Gene Exchanges with the Virulence-Associated *Yersinia pestis* pPCP1 Plasmid

**DOI:** 10.3389/fmicb.2016.00340

**Published:** 2016-03-16

**Authors:** Fabrice Armougom, Idir Bitam, Olivier Croce, Vicky Merhej, Lina Barassi, Ti-Thien Nguyen, Bernard La Scola, Didier Raoult

**Affiliations:** ^1^URMITE, Faculté de Médecine, Centre National de la Recherche Scientifique UM63, CNRS 7257, IRD198, Institut National de la Santé et de la Recherche Médicale 1095, Aix-Marseille UniversitéMarseille, France; ^2^Centre National de la Recherche Scientifique, IRD, Mediterranean Institute of Oceanography, Aix Marseille Université, Université de Toulon, UM110Marseille, France; ^3^Laboratoire VALCORE, Université M'Hamed Bougara BoumerdèsBoumerdès, Algeria

**Keywords:** genomics and evolution, plague pathogenesis, bioinformatics, *Citrobacter koseri*, virulence factors

## Abstract

The history of infectious diseases raised the plague as one of the most devastating for human beings. Far too often considered an ancient disease, the frequent resurgence of the plague has led to consider it as a reemerging disease in Madagascar, Algeria, Libya, and Congo. The genetic factors associated with the pathogenicity of *Yersinia pestis*, the causative agent of the plague, involve the acquisition of the pPCP1 plasmid that promotes host invasion through the expression of the virulence factor Pla. The surveillance of plague foci after the 2003 outbreak in Algeria resulted in a positive detection of the specific *pla* gene of *Y. pestis* in rodents. However, the phenotypic characterization of the isolate identified a *Citrobacter koseri*. The comparative genomics of our sequenced *C. koseri* URMITE genome revealed a mosaic gene structure resulting from the lifestyle of our isolate and provided evidence for gene exchanges with different enteric bacteria. The most striking was the acquisition of a continuous 2 kb genomic fragment containing the virulence factor Pla of the *Y. pestis* pPCP1 plasmid; however, the subcutaneous injection of the CKU strain in mice did not produce any pathogenic effect. Our findings demonstrate that fast molecular detection of plague using solely the *pla* gene is unsuitable and should rather require *Y. pestis* gene marker combinations. We also suggest that the evolutionary force that might govern the expression of pathogenicity can occur through the acquisition of virulence genes but could also require the loss or the inactivation of resident genes such as antivirulence genes.

## Introduction

*Yersinia pestis* is the etiologic agent of plague, a zoonotic disease that led to more than 200 million deaths throughout three pandemic waves (Achtman et al., 2004). It seems that *Y. pestis* has recently evolved from its ancestor *Y. pseudotuberculosis*, usually causing a food-borne gastroenteritis in human (Achtman et al., 2004). Despite their close genetic relationship, which reveals few specific genetic elements, considerable differences subsist in the pathogenicity and transmission mechanisms of *Y. pestis* and *Y. pseudotuberculosis* (Chain et al., 2004).

Following a blood meal from an infected mammalian reservoir, the flea vector colonized by *Y. pestis* in the midgut transmits the pathogen through blood regurgitation during human biting (Perry and Fetherston, 1997). The *Y. pestis* infection cycle involves two acquired plasmids that are absent in the other *Yersinia* species. The 96.2 kb pFra plasmid of *Y. pestis* provides the ability to colonize the flea gut, while the 9.5 kb pPCP1 plasmid (Ferber and Brubaker, 1981; Sodeinde and Goguen, 1988) allows for the dissemination of the bacterium from the peripheral infection site into blood circulation. The pPCP1 plasmid encodes three specific proteins: the bacteriocin pesticin (Pst), the pesticin immunity protein (Pim) and the outer membrane protease (Pla; Hu et al., 1998). The Pla protease plays a central role in the invasiveness of the bubonic plague (Sodeinde et al., 1992), converting plasminogen to plasmin, which dissolves the fibrin clots surrounding the infected site (Bergmann and Hammerschmidt, 2007). However, it has been recently suggested that the first role of the Pla protease is to protect *Y. pestis* from destruction rather to facilitate the bacteria propagation through the host (Guinet et al., 2015). The global threat of the *Y. pestis* pathogen to human health made necessary the development of rapid molecular identification methods that target specific virulence markers, including the detection of the *pla* gene by Polymerase Chain Reaction (PCR) in rodents (Neubauer et al., 2000; Riehm et al., 2011).

Pla belongs to an outer membrane protease family of omptins (Haiko et al., 2009) that supplement the repertoire of potential virulence factors of pathogenic species (Lebrun et al., 2009). The proteolytic activity of omptins causes uncontrolled protein degradation and shifts in regulatory systems that finally drive tissue host disruption, pathogen survival and neutralization of host defense mechanisms (Aepfelbacher et al., 2007). These proteins have been detected in several Gram-negative enteric bacteria and share approximately 50% sequence identity (Haiko et al., 2011). The main members of the family are the OmpT and OmpP of *Escherichia coli* (Stumpe et al., 1998), the PgtE of *Salmonella typhimurium* (Guina et al., 2000), the Pla endopeptidase A of *Erwinia pyrifoliae*, the SopA of *Shigella flexneri* (Egile et al., 1997), and the Pla of *Y. pestis* (Hu et al., 1998).

On June-July 2003, a plague outbreak occurred in the Oran area of Algeria after more than five decades of silence (Bertherat et al., 2007). The Algerian Ministry of Health reported eight bubonic and two septicemia plague cases (Bertherat et al., 2007). One septicemia case was fatal on the outskirts of Oran (Tafraoui). Although any new case has been officially reported since 2003, the continuous surveillance of the Oran region (from 2004 to 2005) resulted in the capture of rodents positively tested for the *Y. pestis pla* gene by rapid molecular method. However, the bacterial species isolated from rodents was a *Citrobacter koseri* strain, not a *Y. pestis* strain as rather expected. *Citrobacter* species are facultative anaerobic Gram-negative bacilli that belong to the *Enterobacteriaceae* family. Among the dozen species of *Citrobacter, C. koseri* is considered as an opportunistic pathogen that is primarily involved in urinary tract infections, neonatal sepsis and meningitis (Kaufman and Fairchild, 2004). In this study, we sequenced and studied the complete genome of our *C. koseri* strain identified in a plague outbreak area. We provided evidence of gene exchanges with the *Y. pestis* virulence pPCP1 plasmid encoding the well-known outer membrane Pla protease.

## Materials and methods

### Rodent site

The rodents were collected from areas where plague cases were reported during the Algeria plague outbreak in 2003; at Kehailia (35°29′N, 0°32′E) and Benaouali (near Zaghloul, (35°33′N, 0°21′E) in the area of Oran and Mascara, ≈450 km West of the capital, Algiers (Bertherat et al., 2007). Rodents were captured inside human residences and from peridomestic areas from September 2004 to May 2005 by using BTS (Besancon Technique Service, INRA, Montpellier, France), Sherman Trap (H.P. Sherman Traps, Tallahassee, FL, USA) and the flooding technique (Blanc and Baltazard, 1945; Pollitzer, 1953). The rodents trapped belong to *Ratus ratus, Ratus norvegicus*, and *Mus spretus* species.

### Ethics statement

Under the supervision of the Algerian Ministry of Health, rodents were caught on areas where confirmed human cases of plague were identified during the 2003 plague outbreak in the region of Oran (Bertherat et al., 2007). The rodents have been collected by Idir Bitam with an official mission order for the surveillance of plague foci (from 2004 to 2005). The Algerian Ministry of Health approved the collection of rodents. No approval of the owners was deemed necessary for entering in private residences to collect rodents. Algeria does not have ethical committee for rodents. No French local Ethic committee reviewed this study. The captured rodents were anesthetized and morphologically identified with the assistance of Kowalsky's work in 1991 (Kowalski and Rzebik-Kowalska, 1991). The rodents were recovered with leather gloves and were anesthetized (Ketamine 40 mg/kg). After the dissection of captured rodents, the recovered spleens were transported in liquid nitrogen to the laboratory and stored at −80° for 15 days before being transported to Marseilles on dry ice.

### Isolation and presumptive identification

The spleens of the rodents were tested for the presence of the *Y. pestis pla* gene by specific molecular-based detection (Charrel et al., 2004). The pla-positive spleen samples were inoculated onto two different culture media. Each spleen was crushed in 500 μl of brain-heart infusion broth, and 100 μl of this broth was inoculated onto both Columbia sheep blood agar (COS, BioMerieux, Marcy l'Etoile, France) and selective *Yersinia* BIN agar plates (Ber et al., 2003). The colonies obtained on both culture media were still tested for *pla* gene by PCR. The pla-positive colonies were checked by 16S rRNA and RpoB gene sequencing. One colony was picked for genome sequencing. The isolate pathogenicity was experimented using a mouse model, preparing a pure culture of the inoculum on COS plates. Colonies were harvested and suspended in sterile distilled water at a concentration of 10^6^/ml, and 100 μl of this suspension was inoculated subcutaneously into four Balb/c mice. The phenotypic profile of the isolate, subcultured on Mac Conkey agar and COP agar plates, was compared with that of the *Y. pestis* EV76 vaccinal strain using API20E strips form BioMerieux (Marcy l'etoile, France), according to the manufacturer's recommendations.

### Genome sequencing

The sequencing of the isolate was carried out through the 454-Roche pyrosequencing (Margulies et al., 2005) and the SOLiD 4 Life technologies. An early shotgun approach was performed with the first-generation 454_GS20. The library was constructed from 5 μg of DNA. Four Picotiterplates were loaded with a 20 Mb capacity each and approximately 100 bp average reads. Next, a paired-end application was performed using the third-generation 454_GSFLX_Titanium. Five μg of DNA was mechanically fragmented on the Hydroshear device (Digilab, Holliston, MA, USA) and visualized through the Agilent 2100 BioAnalyzer on a DNA Labchip 7500, with an optimal size of 3.742 kb. The library was constructed according to the 454 Titanium paired- end protocol and the manufacturer's instructions. The single-stranded paired-end library profile was visualized on an Agilent 2100 RNA Pico 6000 Labchip with the optimal size of 455 bp. The library was quantified on the Quant-it RiboGreen kit (Invitrogen) on the Genios_Tecan fluorometer at 53 pg/μL. The library concentration equivalence was calculated as 2.147E+08 molecules/μL. The library was clonally amplified with one copy per bead in four emPCR reactions with the GS Titanium SV emPCR Kit (Lib-L) v2. Beads were loaded on the GS Titanium PicoTiterplate PTP Kit 70 × 75 and sequenced with the GS Titanium Sequencing Kit XLR70. The quality of the molecule was improved through the SOLiD technology; a paired end library was constructed from 1 μg of purified genomic DNA of the isolate. The sequencing was carried out to 50 × 35 bp using SOLiD™ V4 chemistry on one full slide associated with 92 other projects on an Applied Biosystems SOLiD 4 apparatus. The DNA was fragmented on the Covaris device, and the library concentration was measured on the Qbit fluorometer at 11.4 nmol/L. The libraries were pooled in equimolar ratios, and the size selected on the E-Gel iBase system was between 240 and 270 bp. The ePCR was performed according to the Life Technologies-specific template bead preparation kits on the EZ beads automated Emulsifier, Amplifier and Enricher E80 for full scale. A total of 2,883,835 and 1,176,184 reads were obtained using SOliD and 454 Roche sequencing, respectively.

### Full genome assembly

The data yield by the 454 Roche pyrosequencing were trimmed (1,139,478 trimmed reads) and assembled using both Mira assembler v3.2 (Chevreux et al., 2004) and the Newbler 2.8 software (Roche, 454 Life Sciences). To reduce the dataset, the contigs were combined together by Cisa (Lin and Liao, 2013). Scaffolding was improved using the Opera software v1.2 (Gao et al., 2011) and GapFiller V1.10 (Boetzer and Pirovano, 2012). The repeats, such as rRNA operons, were checked and compared with the current available genome of *C. koseri* ATCC BAA-895 using CLC Genomics software v4.7.2 (CLC bio, Aarhus, Denmark). The genome assembly was improved with manual refinements using the SOLiD run (about 1.5 million of trimmed reads) mapped onto the final assembly. The remaining scaffolds were ordered using the *C. koseri* ATCC BAA-895 reference genome and the MAUVE program version 2.3.1 (Darling et al., 2004). The seventeen gaps between the remaining ordered scaffolds were filled by PCR and several designed primer sets (Untergasser et al., 2012). The circular nature of the plasmids was checked by PCR and by a local python script. The PCR products were sequenced by the Sanger capillarity ABI, and both assembly and sequence integrations were performed using the CAP3 assembler (Huang and Madan, 1999). Finally, five scaffolds were obtained (one bacterial Chromosome and four plasmids). The Genome coverage was 30X using trimmed reads. The *Citrobacter koseri* strain URMITE (CKU) genome has been deposited at the NCBI under the accession number PRJEB6512.

### Genome annotation

The GC-skew diagram was obtained using a GenSkew executable jar file (http://genskew.csb.univie.ac.at). The ORFs were predicted by Prodigal version 2.50 (Hyatt et al., 2010). The ORFs were annotated using BlastP algorithm similarity search against the NCBI Nr database (Benson et al., 2013) and using Rps-Blast against the Pfam database (Punta et al., 2012) of position-specific scoring matrices (PSSM) with *E* < 10^−5^. The functional Clusters of Orthologous Groups (COG) were also identified by Rps-Blast against the COG database (Tatusov et al., 2001) of PSSM with *E* < 10^−5^. The ribosomal rRNA and tRNA were predicted by the RNAmmer 1.2 server (Lagesen et al., 2007) and ARAGORN (Laslett and Canback, 2004). The prophage identification was realized by PHAST (Zhou et al., 2011).

### Comparative genomics

The genomes used for the genomic comparison with CKU were retrieved from the ftp genome project repertoire at the NCBI (https://www.ncbi.nlm.nih.gov/Ftp/). The comparative genomics of the *Citrobacter* species were performed by Get_homologs (Contreras-Moreira and Vinuesa, 2013) to identify the core/pan-genome using the recommendations of Tettelin and collaborators (Lagesen et al., 2007). The software uses the principle of bidirectional best hit, COGsoft (Kristensen et al., 2010) and OrthoMCL v1.4 algorithm (Li et al., 2003). The strain-specific genes are defined as the genes found in only one species and not in all the other *Citrobacter* species of the study. The strain-specific genes were identified by interrogating the pan-genome matrix built by Get_homologs and after removing split genes. The genomic comparison of the Yersiniabactin locus and the conjugative tra operon with their closest matches were realized using the RAST server (Contreras-Moreira and Vinuesa, 2013). The structural mapping was performed using the PYMOL Molecular Graphism System, Schrödinger, LLC and the 2X56 PDBid of *Y. pestis* Pla protein (Eren et al., 2010). The figures were obtained using BRIG (Alikhan et al., 2011), Circos (Krzywinski et al., 2009), Graphlan and Cytoscape 3.0.1 (Saito et al., 2012).

### Phylogenetic analysis of single nucleotide polymorphisms

The kSNP3 program (Gardner et al., 2015) was used to identify pan/core-genome Single Nucleotide Polymorphisms (SNPs) in the set of *Citrobacter* genomes, and to estimate phylogenetic tree based upon those SNPs. The same method was performed on the pCitro2 plasmid with its best blast hits against nr and Whole Genome Shotgun (WGS) databases at the NCBI. The optimal value of K-mer size was obtained by Kchooser of the kSNP3 package. The kSNP3 was performed using the default parameter with the core and the maximum likelihood tree options. The branch lengths are expressed in terms of changes per number of SNPs.

### Phylogenetic analysis of the lpxL gene and omptin family

The *Y. pseudotuberculosis* lpxL gene was blasted against the Nr database. The sequences of the first 50 best hits were retrieved, aligned using MAFFT program (Katoh and Standley, 2013) and curated by Trimal (Capella-Gutierrez et al., 2009). The phylogenetic tree was inferred using MEGA 5 (Gardner et al., 2015) with the maximum likelihood algorithm, bootstrap resampling (500 replicates) and the Kimura2 model. The protein sequences of the omptin family were retrieved from the Pfam web site (http://pfam.sanger.ac.uk/); the same method was applied with the JTT correction for amino acids.

### Phylogenetic analysis of pCitro1 and strain-specific genes

Each of the all predicted proteins was used as seed for BlastP against Nr database. For each protein, the hits that exhibited a significant match (*E* < 10^−4^ and coverage >70%) were selected. Single protein datasets were aligned by MAFFT and trimmed by trimal. One hundred datasets were produced from each dataset by bootstrap resampling using Seqboot in the PHYLIP suite version 3.6 (Felsenstein, J. 2005). Phylogenetic analyses were conducted using the maximum likelihood method with PHYML (Guindon and Gascuel, 2003) and the WAG model (Whelan and Goldman, 2001). The consensus function in PHYLIP was used to construct consensus trees with extended majority rule.

## Results

### Screening rodent tissue and phenotypic features

The spleens of 14 rodents trapped in the Oran area of Algeria (Figure S1), during the surveillance of plague foci, were screened for the *Y. pestis pla* gene by PCR-based method. The *pla* gene was positively detected in five rodent spleens. Each *pla*-positive spleen was therefore inoculated onto both COS and BIN culture media. From the eleven colonies (10 on COS and one on BIN) obtained for the five *pla*-positive spleens, solely two were still positive for the *Y. pestis pla* gene. These two *pla*-positive colonies were obtained on a unique COS plate corresponding to a unique spleen sample (Table 1). The 16S rRNA and RpoB gene sequencing revealed that these two colonies arose from the same bacterium.

**Table 1:** *****Pla***-positive colonies obtained from spleen samples**.

	**Colony number on**		
***pla*-positive sample**	**COS media**	**BIN media**	***pla*-positive colonies**
Spleen1	2	1	0
Spleen2	2	0	0
Spleen3	3	0	0
Spleen4	2	0	**2**
Spleen5	1	0	0

Eleven colonies were obtained on COS and BIN plates from the five pla-positive spleens. As indicated in bold, two pla-positive colonies were identified for the spleen4.

The characteristics of the colonies from our isolate were similar to those of *Y. pestis*, although the colonies from the latter were slightly smaller (Figure S2A). However, the phenotypic profile of our isolate showed major discrepancies with that of *Y. pestis* (Figure S2B). Contrary to *Y. pestis*, our isolate was positive for ONPG and showed the ability to ferment lactose and to use Citrate. Using the BioMerieux database, the biochemical profile of our isolate is finally associated to a *Citrobacter koseri*/*amalonaticus* rather than to a *Y. pestis*. In parallel, the subcutaneous infection of four Balb/c mice with the isolate strain did not produce any pathogenic effects after 2 weeks. Within these contradictions, we carried out the whole genome sequencing of our isolate named *Citrobacter koseri* strain URMITE (CKU). *Citrobacter koseri*, a gram-negative facultative anaerobic bacterium, is an environmental organism commonly found in human and animal gut microbiota, as well as in soil or water (Lin et al., 2011). This species is responsible of central nervous system infections causing sepsis, meningitis and multiple brain abscesses in neonates, young infants or rats (Townsend et al., 2003). However, the pathogenic mechanism is poorly described although the ability to penetrate and survive in macrophage has been reported (Townsend et al., 2003).

### Genome-wide comparison

**Figure 1:**
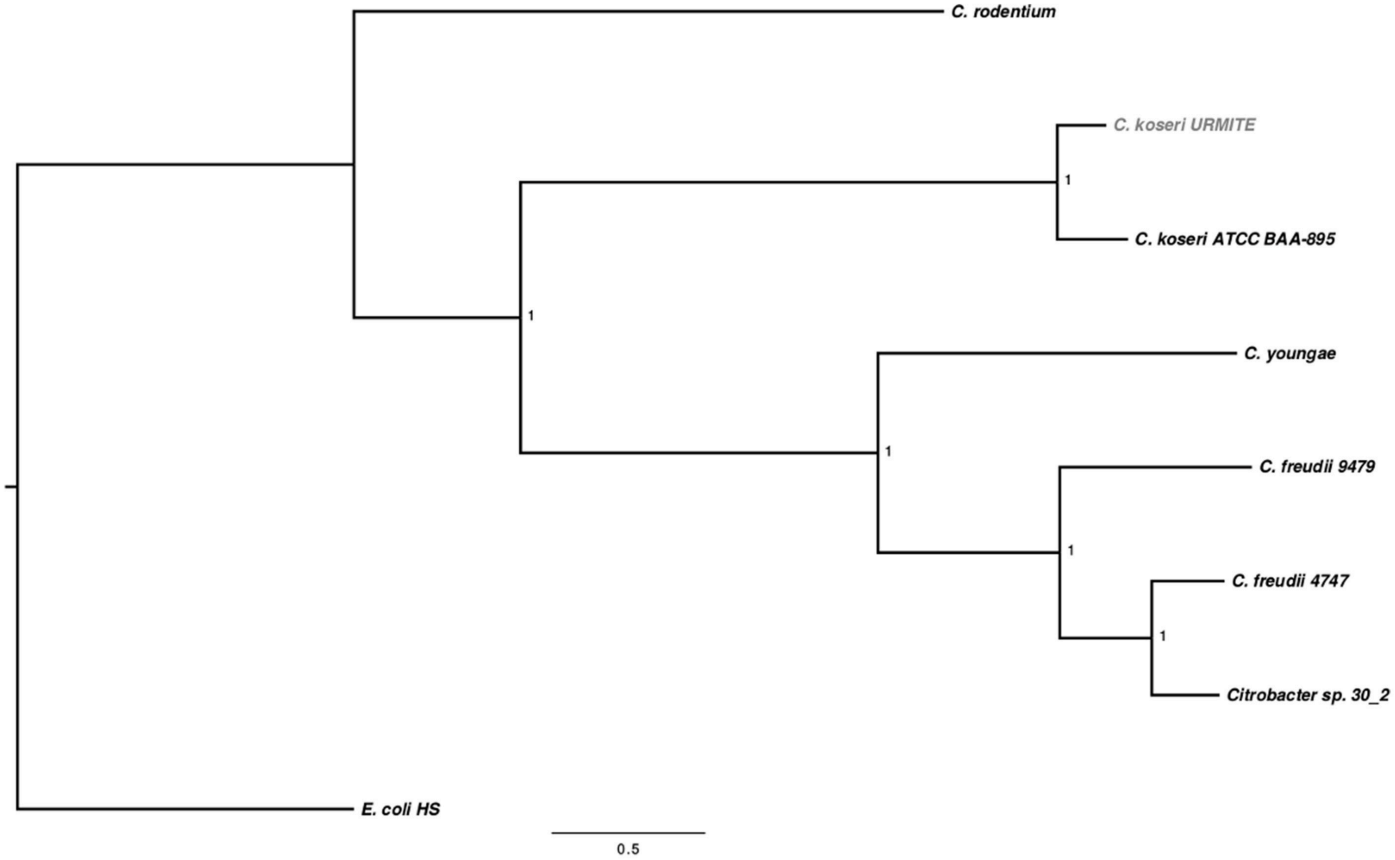
**SNP-based phylogenetic tree of *Citrobacter* species**. SNP-based phylogenetic tree using the SNP data collected from seven *Citrobacter* genomes. The branch supports were indicated as posterior probabilities.

The sequencing of the rodent isolate confirmed the identification of CKU, a circular chromosome of 4,763,705 bp with four circular extra-chromosomal plasmids. The pCitro1 and pCitro2 plasmids are the largest, 170 and 33 kb in size, respectively, and they go along with the smaller pCcitro3 and pCitro4 plasmids, respectively at 3.8 and 5 kb in size. The sequence heterogeneity of the multiple 16S operons of the CKU led us to deduce the phylogenetic relationships using SNP-based core genome variations from several sequenced *Citrobacter* species. The CKU is closely related to the *C. koseri* ATCC BAA-895 strain (Figure 1). In addition, the two strains showed similar functional COG profile (Figure S3).

**Figure 2:**
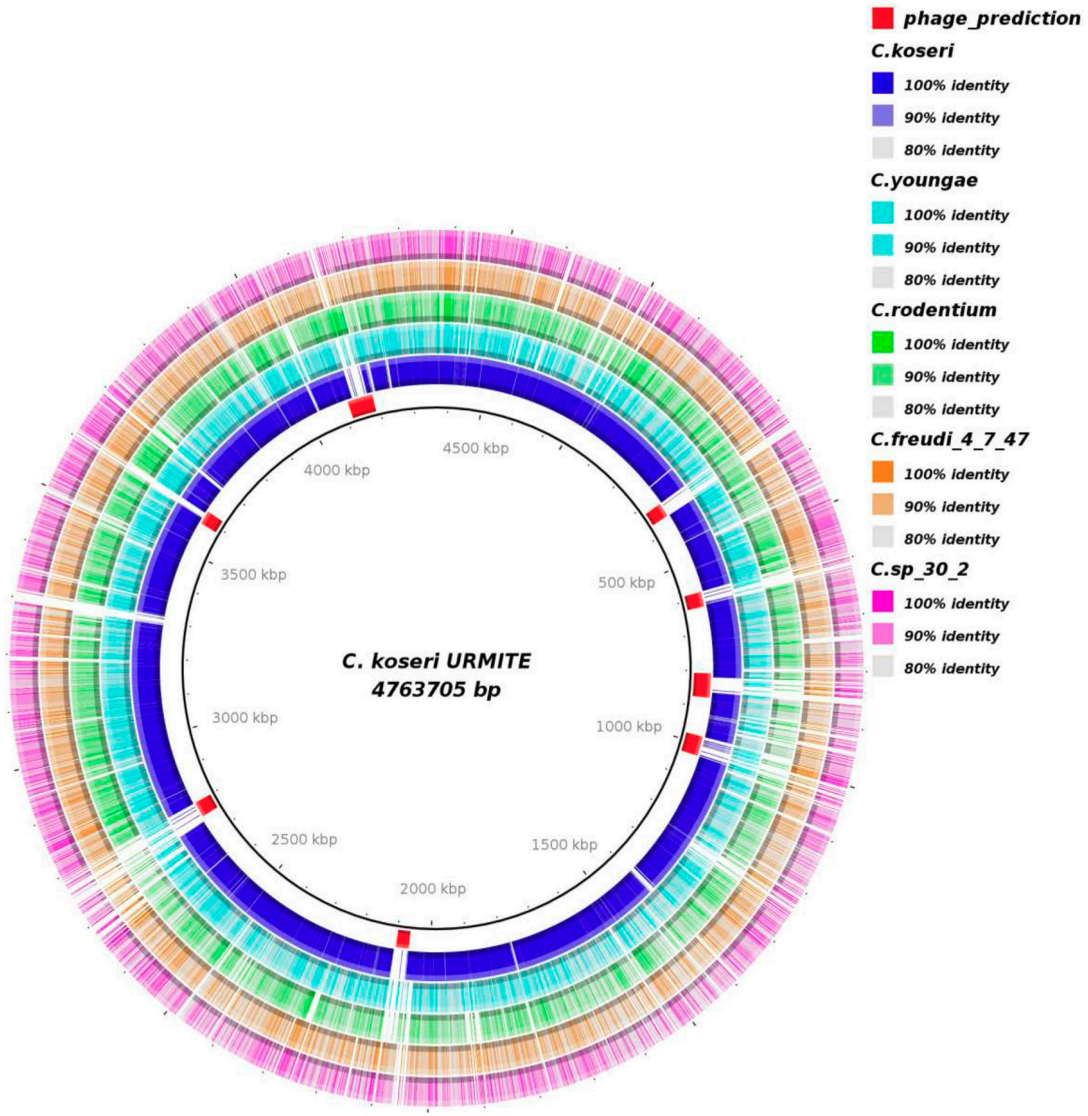
**Genomic comparison of *C. koseri* URMITE with other *Citrobacter* species**. High levels of sequence identity of the *Citrobacter* spp. genomes with the *C. koseri* URMITE genome are indicated as ring with colored tiles/blocks, whereas no or weak sequence identity is shown as white tiles/blocks. The *C. koseri* blue ring corresponds to the *C. koseri* ATCC BAA-895 genome. The *C. koseri* URMITE specific regions are mainly annotated as prophage (red tiles).

A genome-wide comparison of various *Citrobacter* species (Table S1) identified the *C. koseri* ATCC BAA-895 genome as the most similar to our isolate (Figure 2). Interestingly, the Figure 2 also shows specific CKU genomic regions that are missing in the other *Citrobacter* genomes. Most of these regions were predicted as prophages (eight) that spanned approximately 382 kb of the CKU genomic DNA (Table S2, Figure S4). In contrast, the closely related *C. koseri* ATCC BAA-895 strain has only one 35 kb- prophage (related to *E. coli*). Moreover, the CKU genome revealed a region homologous to the Yersiniabactin locus, a ferric iron capture system that belongs to the high pathogenicity island (HPI) of *Y. pestis*. The genomic comparison of the locus showed heterogeneous distribution among several enteric species including *C. koseri* ATCC BAA-895 (Figure S5).

**Figure 3:**
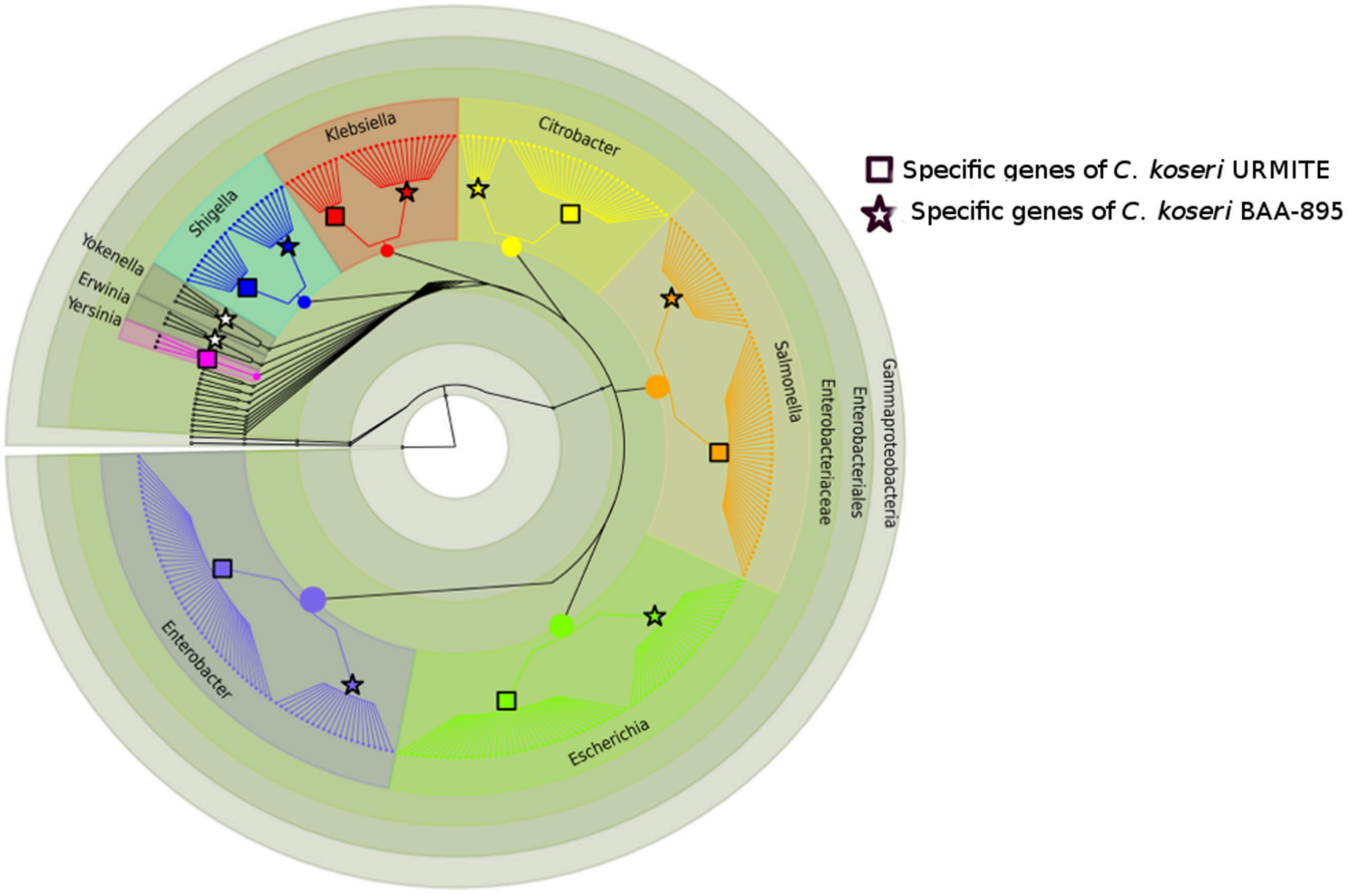
**Phylogeny of strain-specific genes**. The figure indicates the probable phylogenetic origin of the strain-specific genes at the genus level. The *C. koseri* URMITE and *C. koseri* ATCC BAA-895 specific genes are shown by square and star markers, respectively. The gene phylogeny with low branch supports or unresolved were not shown. The flow of gene exchange mainly involved *Enterobacteriaceae* spp.

The comparative genomics of our CKU was also performed at the protein level by a pan-genomic approach with the multiple *Citrobacter* species. The collection of all predicted ORFs of CKU -defined as the orfome- was compared with those collected from *C. koseri* ATCC BAA-895, *C. freudii 4_7_47CFAA, C. freudii GTC 09479, C. youngae* ATCC 29220, *C.* sp 30_2, and *C. rodentium* ICC168 for homolog and recent paralog detection (Table S3). The core-genome shared by all seven species is composed of 2812 ORFs. This core-genome can cover from 58 up to 66% of the orfome of a given *Citrobacter* species. In addition, by interrogating the pangenomic matrix for the dispensable gene content, we reported the strain-specific genes identified in the *Citrobacter* species of the study (Table 2). The *C. koseri* ATCC BAA-895 strain showed 212 specific genes, whereas our CKU strain had 365 specific genes (40% more than *C. koseri* ATCC BAA-895). The *C. rodentium* ICC168 exceeded all the *Citrobacter* species with 937 specific genes. Finally, we compared the phylogenetic origin (genus level) of the specific genes of the closely related *C. koseri* ATCC BAA-895 and URMITE strains. Although a high proportion of the strain-specific genes had an undetermined origin (low boostrap <70 or unresolved trees), the remaining genes that were phylogenically related to *Escherichia, Salmonella, Enterobacter, Klebsiella*, and *Shigella* genera (Figure 3) indicated horizontal gene transfers.

**Table 2:** **Strain-specific genes**.

***Citrobacter* genomes**	**ORF number**	**Specific genes**
*C. koseri* URMITE	4556	365
*C. koseri* BAA895	4269	212
*C. youngae*	4728	489
*C.* sp_30 2	4770	493
*C. freudi* 974	4572	367
*C. freudi* 4747	4848	466
*C. rodentium*	4946	937

The number of strain-specific genes was retrieved from the Citrobacter pangenomic matrix. A strain-specific gene is defined as a gene found in only one species.

### The plasmidome of CKU

**Figure 4:**
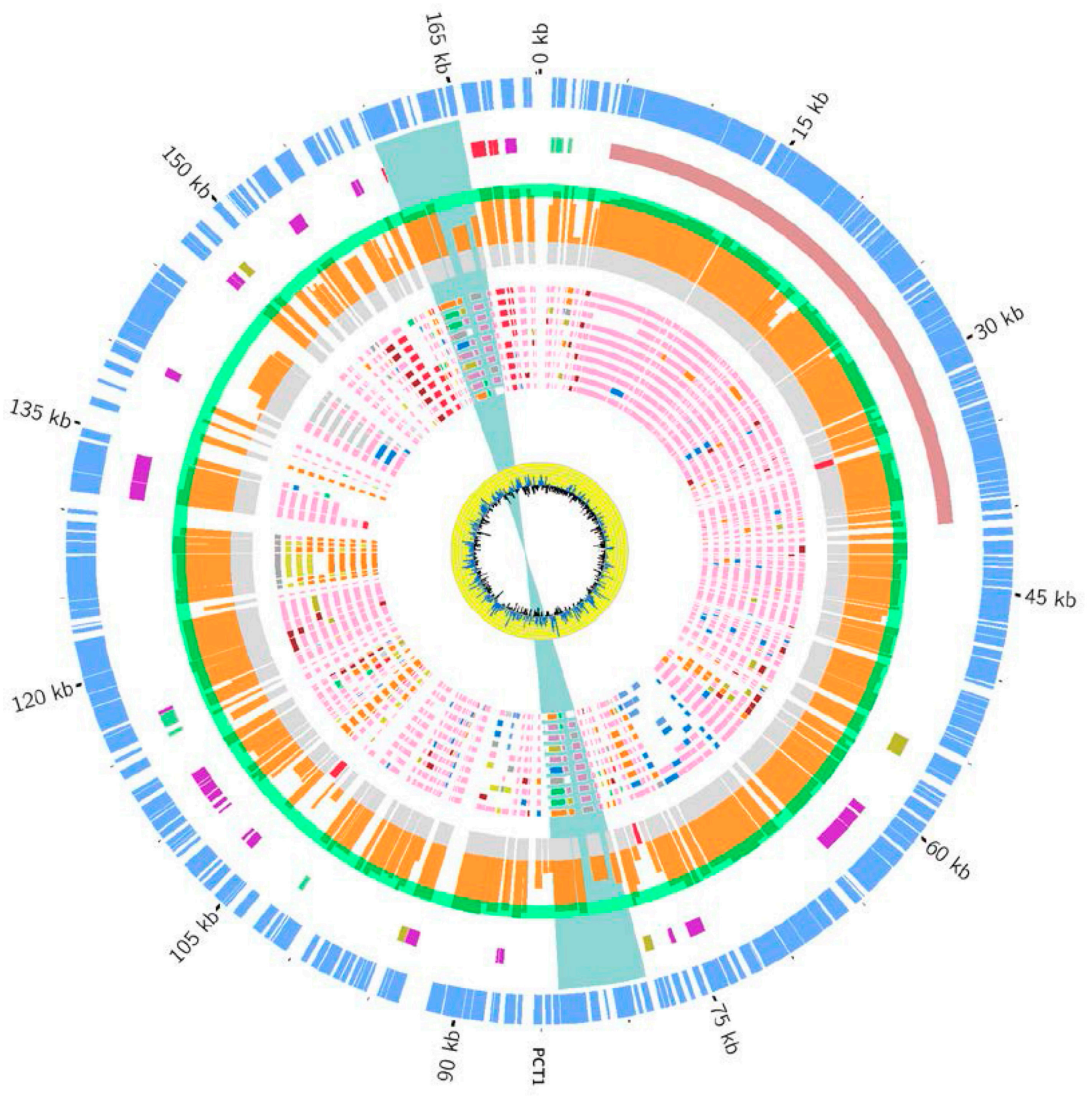
**Overview and organization of the pCitro1 plasmid**. From outside to inside circles. The light blue tiles are the pCitro1 ORFs. The violet tiles are Transposases/Resolvases; the ORFs related to the *Y. pestis* pPCP1 are in red; the tra operon is in dark blue and the blue ribbon indicates a 5 kb inverted repeat. The protein sequence identity with its best blast hit is plotted in the continuous gray (twilight zone <25%), orange (25–90%), and light green (>90%) circles. Below, ten circles showing the taxonomic origin of the ten best blast hits of each protein. Pink, orange, blue and red tiles belong to *Escherichia, Citrobacter, Klebsiella* and *Yersinia* genera, respectively. The yellow circle plot is the GC skew.

The CKU strain contains four plasmids with very specific features. The pCitro1, a large circular plasmid of 170,167 bp with a GC content of 48.07%, is unique to the CKU strain. The annotation of the 213 predicted ORFs led to the identification of 202 proteins that can be classified into 5 functional categories: the replication system, the stabilization and repartition system, the conjugation system and the potential virulence elements (Figure 4, Table S4). Among the potential replicon systems, one is similar to the region previously reported for the R100 plasmid, including the Replication initiator protein, the CopB regulator and a sequence with strong sequence identity (93%) with the unique 149 bp sequence of the replication origin. In addition, an arsenal of stabilization systems for post-segregation killing of plasmid-free cells was predicted, including three families of toxin-antitoxin modules with the CcdAB, PemLK, and RelBE systems. Finally, the pCitro1 plasmid contains a putative conjugation-related region of 36 kb (Table S4) closely related to those of *E. coli* plasmids (Figure S6) and a 19 bp transfer initiation.

The 33-kb pCitro2 plasmid exhibited the best sequence identity (98%) and coverage (83%) with a genomic fragment present in multiple Peruvian *Y. pestis* draft genomes as well as with several *Enterobacteriaceae* plasmids (97–96% of identity and 83% of coverage) including *Enterobacter* spp., *Klebsiella* spp., *C. freudii*, and *S. marcescens* species. No other significant hits were identified. The SNP-based phylogeny tree built with the best hits indicated that pCitro2 was more closely related to the phylogenetic group composed by *E. asburiae*/*cloacae* and *S. marcescens* plasmids rather than with the fragments of the Peruvian *Y. pestis* strains (Figure S7). The *S. marcescens* plasmid has been isolated from human bronchial aspirate in Mexico, while the *E. asburiae* and *E. cloacae* plasmids have been isolated from human stool samples in United States of America (USA). The pCitro2 plasmid contains 39 ORFs encoding for the replication initiation, the post-segregation system and the VirB conjugal transfer family (Table S5). The proteins have best sequence similarity with proteins of multiple *Enterobacteriaceae* species including those of the Peruvian *Y. pestis* strains. The last two small plasmids are mainly composed of hypothetical proteins.

### The plasmidome of CKU reveals exchanges with *Yersinia pestis*

#### A mosaic structure

**Figure 5:**
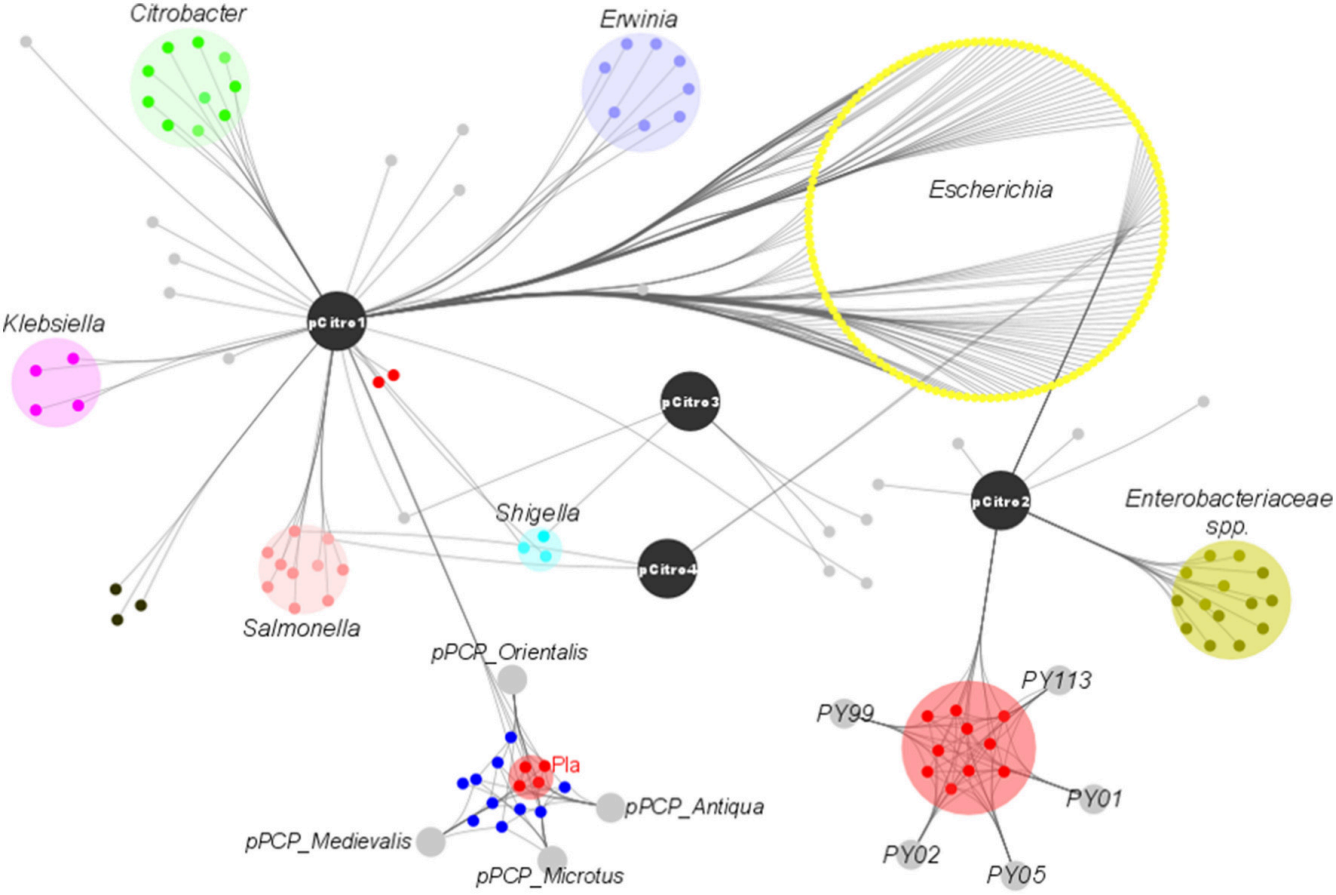
**The taxonomic diversity of the plasmidome**. Protein origins of the plasmidome using phylogenetic inference or best Blast hit. The pCitro1 proteins were related to multiple genera of the *Enterobacteriaceae* group including *Klebsiella, Salmonella, Erwinia, Escherichia, Citrobacter* and *Yersinia*. Four proteins of the pCitro1, including Pla, are exclusively shared with the virulence-associated 9.6 kb *Y. pestis* pPCP1 plasmid biovar Antiqua, Orientalis, Microtus, and Medievalis. The pCitro2 proteins were related to the *Enterobacteriaceae* group including the *Yersinia* genus. Several proteins of pCitro2 are identified as homologs to those from PY99, PY113, PY02, PY05, PY01 contigs of Peruvian *Y. pestis* species. We showed only five members of these *Y. pestis* strains but more than 50 Peruvian *Y. pestis* draft genomes exhibit a genomic fragment highly similar to the complete sequence of pCitro2.

The phylogenetic assignment at the genus level revealed that the predicted proteins of pCitro1 were related to the *Enterobacteriaceae* familly including *Escherichia* spp. (30%), *Citrobacter* spp., *Yersinia* spp.*, Salmonella* spp., *Shigella* spp., *Klebsiella* spp., and *Erwinia* spp. In addition, most of the predicted proteins of the pCitro2 were related to either *Escherichia* spp., *Enterobacteriaceae* spp., or *Yersinia* spp. The plasmidome of CKU finally showed a complex and diverse taxonomic origin of its protein repertoire (Figure 5) that could result from a sympatric lifestyle of the isolate.

#### Exchange with the virulence-associated *Y. pestis* pPCP1 plasmid

**Figure 6:**
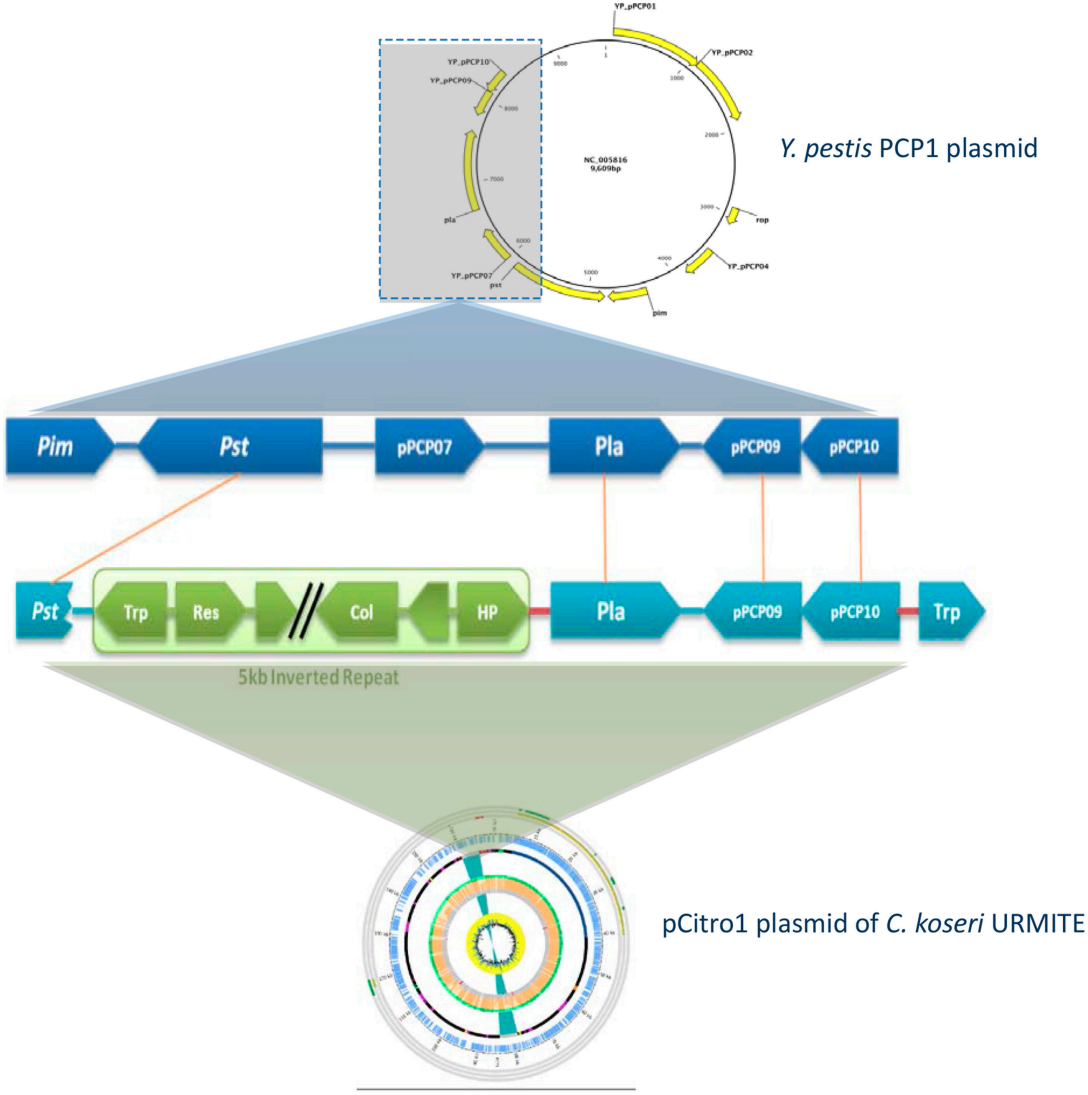
**Comparison of the *pla* region of *Y. pestis* pPCP1 with the pCitro1**. The 9.6 kb *Y. pestis* pPCP1 plasmid shows major similarity points with a pCitro1 region. The structural organization and the protein sequence identity (>99%) are highly conserved between pCitro1 and *Y. pestis* pPCP1 for Pla, PCP09 and PCP10 proteins. The PCP07 and the immunity Pim protein were not found in pCitro1. A fragment (54 amino acids) of the specific bacteriocin Pst protein is recovered downstream the 5 kb inverted repeat of pCitro1. Trp, Res and HP indicate transposase, resolvase, and hypothetical proteins, respectively.

**Figure 7:**
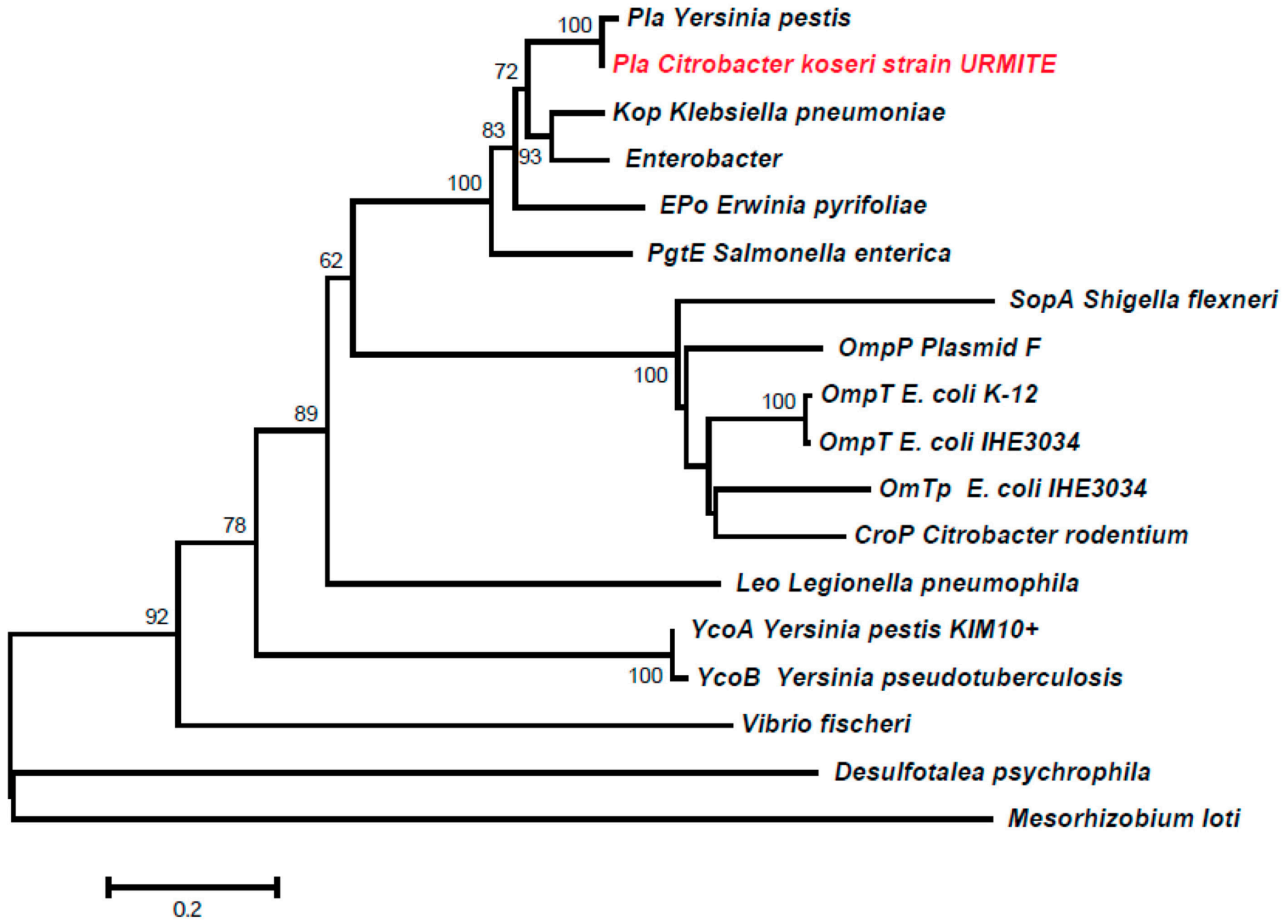
**Phylogeny inference of the main members of the omptin family including the predicted Pla protein of pCitro1**. The phylogeny of the omptin family indicated that our predicted Pla protein is closely related to that of *Y. pestis*.

A 5 kb inverted repeat containing resolvases and transposases intervenes two DNA fragments of pCitro1 that show high sequence identity level with a major part of the virulence-associated *Y. pestis* pPCP1 plasmid (Figures 5, 6). The first short DNA fragment (300 bp) is similar to the pPCP1 region encoding the Pesticin bacteriotoxin (Pst), a glucosaminidase that degrades murein. While the Pst protein of pPCP1 is 357-amino-acids long, the pCitro1 homolog protein is only 54-amino acids long. It is known that Pesticin has no homology to any other bacteriocin of the *Enterobacteriaceae* group. The putative incomplete Pst protein of pCitro1, with 98.15% of residue identity (Townsend et al., 2003; Lin et al., 2011), is therefore closely related to that of *Yersinia pestis* (no other significant hits). In addition, downstream to the 5 kb inverted repeat, a second long DNA fragment (~1.9 kb) shares strong sequence identity with the pPCP1 plasmid of several *Y. pestis* spp. (> 98%) and raises 98.91% of identity with that of the *Y. pestis* biovar Microtus. This region encodes 3 continuous proteins (Figure 6): the plasminogen activator Pla (YP_pPCP08), a putative transcriptional regulator (YP_pPCP09) and a hypothetical protein (YP_pPCP10). These pPCP1 proteins are remarkably conserved in the pCitro1. Indeed, the proteins YP_pPCP09 and YP_pPCP10 are completely identical to the corresponding pCitro1 ORF208 and ORF209, while the plasminogen activator (YP_pPCP08) protein shares 99.36% of sequence identity with the ORF210 of pCitro1. There are only two non-synonymous mutations in our plasminogen Pla predicted protein. These two mutated residues, Val (Leu 51 in Pla of *Y. pestis*), and Gly (Val 185 in Pla of *Y. pestis*), are nearby the residues of the catalytic site when the three-dimensional structure of the Pla protein folding is considered (Figure S8). Finally, the phylogenic relationships with the main members of the omptin protein family confirmed that our predicted Pla is more closely related to *Y. pestis* than it is to *Citrobacter rodentium* (Figure 7). With the exception of the Pla protease, no other omptin protease was identified in the CKU genome.

## Discussion

The acquisition of the pFra and pPCP1 plasmids has been essential for the evolution of *Y. pestis* from *Y. pseudotuberculosis* (Rajanna et al., 2010). These acquired plasmids play a key role in the infection cycle of *Y. pestis* although their origin and mobility remain poorly understood mainly because of limited sequence similarity with other plasmids (Whelan and Goldman, 2001). However, the specific pFra plasmid which carries some potential virulence factors facilitating the *Y. pestis* colonization of the flea midgut has shown evidence of gene exchange with the human pathogen *Salmonella enterica* serovar Typhi (Whelan and Goldman, 2001). On the contrary, the second specific pPCP1 plasmid which carries Pla, an invasion-promoting protease, is considered as unique to *Y. pestis* (Lin et al., 2011). This theory seems to vanish based on our findings and those described in a recent short report (Janse et al., 2013). In their work, Janse and colleagues identified a short sequencing fragment related to the *Y. pestis pla* gene. However, this sequencing fragment was flanked by genomic regions that were not related to those of the *Y. pestis* pPCP1 plasmid but rather to a replicon system (Janse et al., 2013). Contrary to Janse and colleagues findings, our *pla* gene carried by the pCitro1 is complete and located in a continuous genomic region of approximately 1900 bp which encodes 3 proteins that are remarkably conserved in their structural organization and sequence identity with those of the *Y. pestis* pPCP1 (Figure 6). These findings clearly indicate that a single gene or gene block can be horizontally transferred from the *Y. pestis* pPCP1-associated virulence genes to an *Enterobacteriaceae* species and vise versa. As such, the use of the *pla* virulence gene as a single gene maker for the molecular detection of plague becomes inadequate (Higgins et al., 1998; Loiez et al., 2003; Adjemian et al., 2008). The development of rapid and easy detection of plague by Pla-specific monoclonal antibodies should require cross-reaction tests with the homolog Pla protease of *C. koseri* URMITE (Simon et al., 2013). The studies focusing only on *pla*-positive detection do not provide any proof on the presence of *Y. pestis* (Ziwa et al., 2013). Reciprocally, a question subsists for our 4 *pla*-positive spleens. As *pla*-negative colonies were obtained for these spleens, we cannot exclude that these spleens were not infected by *Y. pestis*. The molecular detection of other gene markers of *Y. pestis* and/or *C. koseri* would have resolved this ambiguity. Overall, the molecular identification tools targeting solely the *pla* gene provide a rapid diagnosis but could increase the false positive rate of plague cases. Consequently, the surveillance of plague foci should require the identification of gene marker combinations (Janse et al., 2010). Comparative genomics in the current massive sequencing period could assist in identifying new gene markers of *Y. pestis*.

The virulence-associated bacterial genes are assumed to be carried by mobile genetic elements including plasmids, phages or pathogenicity islands (PAI; Pallen and Wren, 2007). As such, the CKU genome contains the well-known PAI Yersiniabactin siderophore (Figure S5), an iron uptake system that is required for the multiplication of the bacteria in eukaryotic hosts (Perry and Fetherston, 2011). Initially found in *Yersinia* spp., this iron uptake system is now widely distributed among other *Enterobacteriaceae* species, including *Citrobacter* spp. (Carniel, 2001). Moreover, the CKU plasmidome shares several ortholog proteins (including Pla) with the virulence-associated *Y. pestis* pPCP1 plasmid, as well as homologs to VirB proteins that belong to the bacterial type IV secretion system. Although the acquisition of virulence-associated genes might have driven pathogenicity of the CKU strain; it was not the case in our experimental conditions. As we initially believed that our CKU was a *Y. pestis* strain, we performed a virulence experiment commonly applied for *Y. pestis* strains, which consisted in the subcutaneous injection of the strain in mice for mimic the fleabite. However, this primary experimental result should be confirmed by other virulence experiments to conclude about the non-pathogenicity of our CKU strain for animals. Indeed, the expression of pathogenicity can be host-specific and could depend on the infection routes.

Nonetheless, our findings and genomic elements of the CKU could lead to wonder about the expression of virulence factors. The virulence nature of the *pla* gene was initially demonstrated using *Y. pestis* isogenic strains (Sodeinde et al., 1992). On the contrary, it has been reported that expression of the *Y. pestis* pPCP1 plasmid did not significantly influence the *Y. pseudotuberculosis* virulence (Kutyrev et al., 1999) or that wild-type *Y. pestis* strains lacking the pPCP1 plasmid were still virulent (Samoilova et al., 1996; Welkos et al., 1997). The transformation of an virulent strain with a virulence gene does not necessarily lead to pathogenicity. This suggests that additional genetic factors may be required (Sanders et al., 1997) or that resident genes may diminish the expression of this pathogenicity (Bliven and Maurelli, 2012). In many cases, the function and the phenotype expression of newly captured virulence genes, by commensal or pathogenic bacteria, can be blocked or reduced by some resident genes, aptly named “antivirulence genes” (Bliven and Maurelli, 2012). Interestingly, comparative genomics of the CKU strain identified a homolog of the antivirulence *lpxL* gene (Bliven and Maurelli, 2012) that is lacking in all sequenced *Y. pestis* strain (Montminy et al., 2006; Table S6, Figure S9). The *lpxL* gene encodes an acyltransferase that modifies the bacterial lipopolysaccharide (LPS) and provokes a LPS-induced inflammatory response. The expression of a functional *lpxL* in a *Y. pestis* isogenic strain induced an appropriate immune response in a mouse model without any sign of disease. In contrast, the non-functional *lpxL* wild-type strain resulted in 100% mortality (Montminy et al., 2006). Through the inactivation of the *lpxL* gene, the highly pathogenic *Y. pestis* optimized its pathogenicity and increased its ability to evade host immune responses (Bliven and Maurelli, 2012). In this way, the presence of an *lpxL* gene in the CKU strain could be one of the critical factors that interfere with the pathogenicity expression within mammals.

The genome and mobilome of CKU exhibited massive gene exchanges with closely related enteric species (Figures 3, 5). The lifestyle of CKU shapes its genome evolution and plasticity, contributes to the bacterial diversification that might redefine ecological niches and promotes bacterial speciation. We foresee this is one of the main evolution ways for specialized pathogenic bacteria. In sympatric lifestyles, new gene repertoires are permanently created through the integration of foreign DNA. Some of those repertoires may endow bacteria with the ability to adapt to a specific niche, and this may be fulfilled by the deletion or the inactivation of non-virulence and antivirulence genes, such as observed for *Shigella* or *Y. pestis*. As a result, zoonoses might represent a potential reservoir for the development of future pathogens.

## Author contributions

DR conceived the project, wrote the paper. BL conceived the project, wrote the paper. FA wrote manuscript, performed bioinformatics analysis, performed experiments. IB collect the rats sample, wrote the paper. OC performed bioinformatics analysis. VM performed bioinformatics analysis. TN performed experiments. LB performed experiments.

### Conflict of interest statement

The authors declare that the research was conducted in the absence of any commercial or financial relationships that could be construed as a potential conflict of interest.
