# Supplementary material for: Genomic Insights into a New Citrobacter koseri Strain Revealed Gene Exchanges with the Virulence-Associated Yersinia pestis pPCP1 Plasmid
Source: Front Microbiol. 2016 Mar 16;7:340. doi: 10.3389/fmicb.2016.00340 (PMC4793686; doi:10.3389/fmicb.2016.00340)
Supplement: Supplementary file 1 [file Table1.PDF]

Table S1: Genomic features of Citrobacter spp. genomes

Species	Genome size (bp)	GC %	Status	Accession number
C. freudii 4_7_47	5,120,174	52.59	Draft	PRJNA80411
C. rodentium ICC168	5,346,659	54.72	Complete	NC_013716
C. koseri ATCC BAA 985	4,720,462	53.83	Complete	NC_009792
C. koseri URMITE	**4,763,704**	**53.84**	**Complete**	**PRJEB6512**
C. youngae ATCC 29220	5,150,259	52.23	Draft	PRJNA5508
C. sp. 30_2	5,122,674	52.61	Draft	PRJNA55595
C. freudii GTC 0974	4,899,578		Draft	PRJNA187423
