# Supplementary material for: Genomic Insights into a New Citrobacter koseri Strain Revealed Gene Exchanges with the Virulence-Associated Yersinia pestis pPCP1 Plasmid
Source: Front Microbiol. 2016 Mar 16;7:340. doi: 10.3389/fmicb.2016.00340 (PMC4793686; doi:10.3389/fmicb.2016.00340)
Supplement: Supplementary file 2 [file Table2.PDF]

Table S2: Prophage prediction in the *C. koseri* URMITE chromosome

Region	Size (kb)	GC%	CDS	Phage hits	Possible prophage Accession number	Completeness
1	38.1	53.69	59	44	Burkholderia BcepMu (NC_005882)	Questionnable
2	40.4	54.22	45	32	Bordetella BPP-1 (NC_005357)	Intact
3	65.9	52.13	68	54	Enterobacteria phiP27 (NC_003356)	Intact
4	53.5	50.55	88	74	Salmonella SPN3UB (NC_019545)	Intact
5	42.8	50.34	64	45	Salmonella Fels-1 (NC_010391)	Intact
6	42	53.36	46	32	Acyrthosiphon pisum APSE-1 (NC_000935)	Intact
7	14.1	50.00	27	15	Salmonella Fels-1 (NC_010391)	Incomplete
8	70.6	52.78	62	44	Enterobacteria fiAA91_ss (NC_022750)	Intact

General features of the predicted prophage regions. The prophage completeness is assessed by PHAST score scheme. The possible prophage assignment corresponds to the phage with the highest number of predicted protein hits.
