# Supplementary material for: Genomic Insights into a New Citrobacter koseri Strain Revealed Gene Exchanges with the Virulence-Associated Yersinia pestis pPCP1 Plasmid
Source: Front Microbiol. 2016 Mar 16;7:340. doi: 10.3389/fmicb.2016.00340 (PMC4793686; doi:10.3389/fmicb.2016.00340)
Supplement: Supplementary file 3 [file Table3.PDF]

Table S3: Orthologs and in-paralogs detection of pairwise species

Citrobacter spp.	CKU	freudii_4_7_47	freudii_9479	koseri BAA895	rodentium	youngae	sp. 30_2	In-paralogs
**CKU**	_	3480	3502	**4031**	3426	3456	3419	142
**freudii_4_7_47**	3480	_	3973	3501	3363	**3907**	**4157**	106
**freudii_9479**	3502	3973	_	3562	3428	3953	3999	93
**koseri BAA895**	**4031**	3501	3562	_	3387	3542	3497	113
**rodentium**	3426	3363	3428	3387	_	3454	3400	**1154**
**youngae**	3456	3907	3953	3542	**3454**	_	3870	134
**sp. 30_2**	3419	**4157**	**3999**	3497	3400	3870	_	100

Orthologs and in-paralogs identification using Get_homologues and best reciprocical blast hit with a minimum of 50 % of sequence identity and coverage.
