# Supplementary material for: Genomic Insights into a New Citrobacter koseri Strain Revealed Gene Exchanges with the Virulence-Associated Yersinia pestis pPCP1 Plasmid
Source: Front Microbiol. 2016 Mar 16;7:340. doi: 10.3389/fmicb.2016.00340 (PMC4793686; doi:10.3389/fmicb.2016.00340)
Supplement: Supplementary file 4 [file Table4.PDF]

Table S4: Replication, maintenance, partitioning and conjugative systems of the pCitro1 plasmid.

ORFs	Replication System	Maintenance/ Partition Systems	Conjugation System	*Yersinia pestis* proteins
ORF1-3	RepA/RepA1/CopB			
ORF4		Hok/gef		
ORF9-50			Operon Tra	
ORF55-56		**PsiA/PsiB**		
ORF72-73		**Par type II**<br>**StbA/StbB**		
ORF99-100		PemI/PemK		
ORF108		**ParA-like**		
ORF116-117		CcdB/CcdA		
ORF127	RepA			
ORF143-144	RepA/RepA1/CopA			
ORF153-158<br>ORF165-168			Pili assembly	
ORF171-172		StbE/StbD		
ORF187				Pesticin (Fragment)
ORF193				Plasminogen protease
ORF194				Transcriptional Regulator
ORF195				Hypothetical protein

Major replication, maintenance, partitioning (in bold) and conjugative systems found in pCitro1
