# Supplementary material for: Genomic Insights into a New Citrobacter koseri Strain Revealed Gene Exchanges with the Virulence-Associated Yersinia pestis pPCP1 Plasmid
Source: Front Microbiol. 2016 Mar 16;7:340. doi: 10.3389/fmicb.2016.00340 (PMC4793686; doi:10.3389/fmicb.2016.00340)
Supplement: Supplementary file 5 [file Table5.PDF]

Table S5: Annotation of the pCitro 2 plasmid

ORF	Cover	ID	Hit description	BBH	Phylogeny (bootstrap >70)
1	100	94	bacterial conjugation TrbI-like protein	*Enterobacteriaceae*	*undetermined*
2	100	99	integrating conjugative element PilL	*Y. pestis& other Enterobacteriaceae*	*undetermined*
3	100	97	transglycosylase SLT domain protein	*Enterobacteriaceae*	*undetermined*
4	100	97	hypothetical protein G716_04885	*Enterobacteriaceae*	*undetermined*
5	100	99	plasmid replication initiator RepA	*Enterobacteriaceae*	*undetermined*
6	100	100	Putative DNA-invertase resolvase	*E. coli*	*E. coli*
7	100	90	transposase for transposon	*E. coli*	*E. coli*
8	100	96	hypothetical protein	*S. marcescens*	*S. marcescens*
9	100	99	hypothetical protein	*Enterobacteriaceae*	*undetermined*
10	30	48	hypothetical protein	*Enterobacteriaceae*	*Undetermined*
11	100	100	chaperonin ybiA	*Y. pestis& other Enterobacteriaceae*	*Undetermined*
12	100	98	ATPase MipZ family protein	*Y. pestis& other Enterobacteriaceae*	*Undetermined*
13	100	99	antirestriction familly protein	*S. marcescens*	*Undetermined*
14	100	98	phage integrase familly protein	*S. marcescens*	*S. marcescens/E.coli*
15	100	89	hypothetical protein	*E. coli*	*E. coli*
16	100	95	hypothetical protein	*E. coli*	*E. coli*
17	35	59	hypothetical protein	*E. coli*	*E. coli*
18	100	98	post segregation antitoxine CcdA	*E. coli*	*undetermined*
19	100	98	putative transcriptionnal regulator	*E. coli*	*E. coli*
20	100	97	plasmid stabilization ParB	*E. coli*	*E. coli*
21	100	100	hypothetical protein	*E. coli*	*E. coli*
22	100	100	hypothetical protein	*Y. pestis*	*undetermined*
23	100	99	antirestriction protein family	*E. coli*	*undetermined*
24	100	99	hypothetical protein	*Y. pestis*	*Undetermined*
25	100	100	hypothetical protein	*Enterobacteriaceae*	*Undetermined*
26	100	100	ParB-like protein	*Enterobacteriaceae*	*Undetermined*
27	100	99	hypothetical protein	*Y. pestis*	*Y. pestis*
28	100	98	hypothetical protein	*Y. pestis*	*Y. pestis*
29	100	100	type II/IV secretion system protein	*Y. pestis*	*undetermined*
30	52	38	hypothetical protein	*l. cohaerens*	*undetermined*
31	98	100	hypothetical protein	*Y. pestis*	*Y. pestis*
32	100	99	hypothetical protein	*Y. pestis*	*Y. pestis*
33	100	100	ATPase AAA	*Y. pestis*	*undetermined*
34	100	99	hypothetical protein	*Y. pestis*	*Y. pestis*
35	100	98	hypothetical protein	*Y. pestis/E. cloacae*	*undetermined*
36	99	98	trbL/VirB6 plasmid conjugal transfer protein	*Y. pestis*	*undetermined*
37	100	99	trbL/VirB6 plasmid conjugal transfer	*Enterobacteriaceae*	*undetermined*
38	100	99	virB8 conjugal transfer family protein	*Enterobacteriaceae*	*undetermined*
39	100	99	conjugal transfer family protein Virb9/TrbX	*Y. pestis& other Enterobacteriaceae*	*undetermined*

Id, cover and BBH indicate sequence identity, sequence coverage and best blast hit, respectively. The “Undetermined” tag was used for low branch support.
