# Supplementary material for: Genomic Insights into a New Citrobacter koseri Strain Revealed Gene Exchanges with the Virulence-Associated Yersinia pestis pPCP1 Plasmid
Source: Front Microbiol. 2016 Mar 16;7:340. doi: 10.3389/fmicb.2016.00340 (PMC4793686; doi:10.3389/fmicb.2016.00340)
Supplement: Supplementary file 6 [file Table6.pdf]

Table S6: **Screening of *Yersinia pestis* antivirulence genes in CKU**

***Yersinia* antiviruence genes**	**Accession number**	**E-value**	**Identity (%)**	**Coverage (%)**
rcsA_LuxR_family_transcriptional_regulator	YP_070999	$2\ 10^{-4}$	33.93	28.9
nghA_beta-N-acetylhexosaminidase	YP_069661	6.4	34.25	7.0
lipid_A_biosynthesis_lauroyl_acyltransferase (lpxL)	**CAH21728**	**$2.5\ 10^{-13}$**	**59.60**	**98.7**

Three antivirulence genes lost by *Yersinia pestis* spp. were used for homology detection in the CKU genome using Blast algorithm. The antivirulence proteins were retrieved from *Y. pseudotuberculosis* IP32953 genome. The E-value, sequence identity and coverage represent best blastp hit scores against the CKU proteome.
