# Supplementary material for: Genomic Insights into a New Citrobacter koseri Strain Revealed Gene Exchanges with the Virulence-Associated Yersinia pestis pPCP1 Plasmid
Source: Front Microbiol. 2016 Mar 16;7:340. doi: 10.3389/fmicb.2016.00340 (PMC4793686; doi:10.3389/fmicb.2016.00340)

Supplementary Figure S1: Plague area

A. Sites of rodent collection in the Oran area of the Algerian plague outbreak

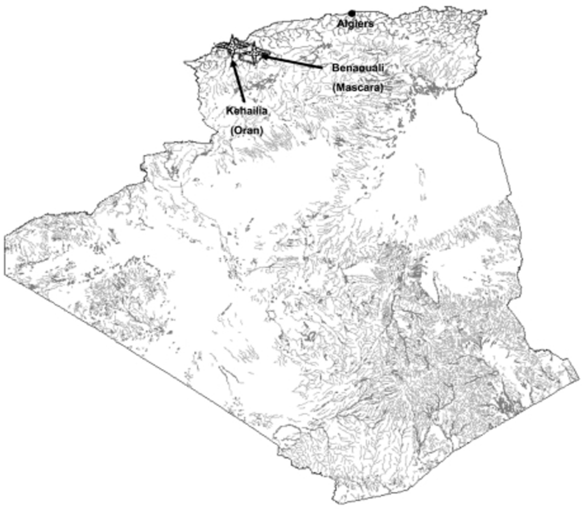

B. Field and rodents trapping

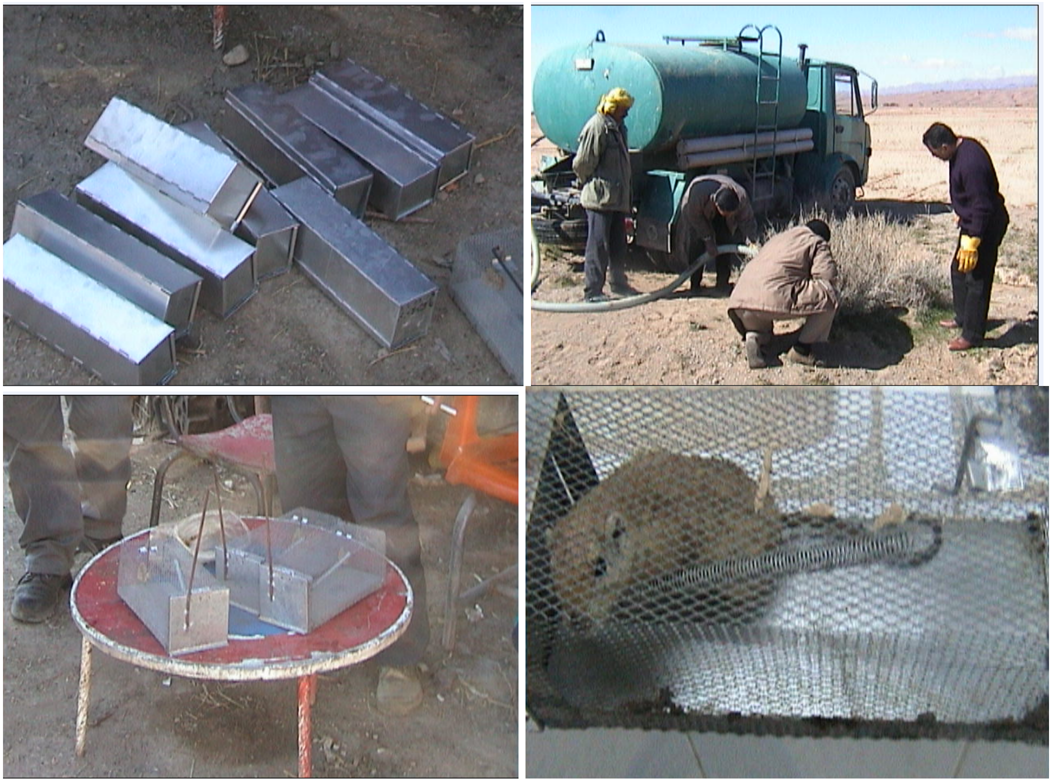

Supplement: Supplementary file 7 [file Image1.PDF]
