# Supplementary material for: Genomic Insights into a New Citrobacter koseri Strain Revealed Gene Exchanges with the Virulence-Associated Yersinia pestis pPCP1 Plasmid
Source: Front Microbiol. 2016 Mar 16;7:340. doi: 10.3389/fmicb.2016.00340 (PMC4793686; doi:10.3389/fmicb.2016.00340)

**Figure S2: Comparison of our isolate with *Y. pestis***

**a.** Aspect of the bacteria colonies from our *C. koseri* URMITE isolate (left) and from *Y. pestis* (right) using Columbia sheep blood agar.

**b**. Results of the APIE strip of our *C. koseri* URMITE (below) isolate and *Y. pestis* (above)

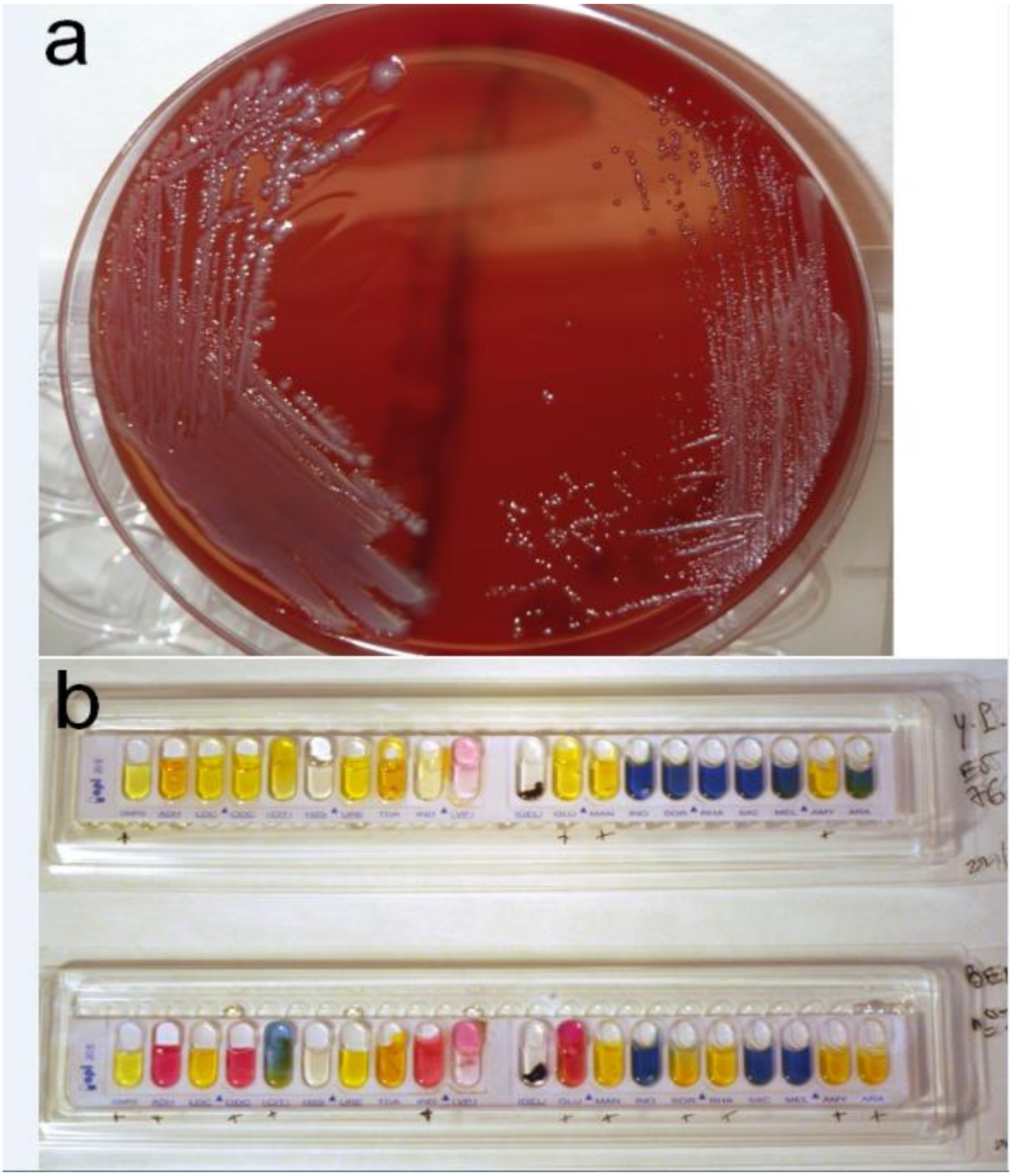

Supplement: Supplementary file 8 [file Image2.PDF]
