# Supplementary figures and images for: Genomic Insights into a New Citrobacter koseri Strain Revealed Gene Exchanges with the Virulence-Associated Yersinia pestis pPCP1 Plasmid
Source: Front Microbiol. 2016 Mar 16;7:340. doi: 10.3389/fmicb.2016.00340 (PMC4793686; doi:10.3389/fmicb.2016.00340)

**Figure S3: Distribution of functional COGs in *C. koseri* ATCC BAA-895 and CKU**

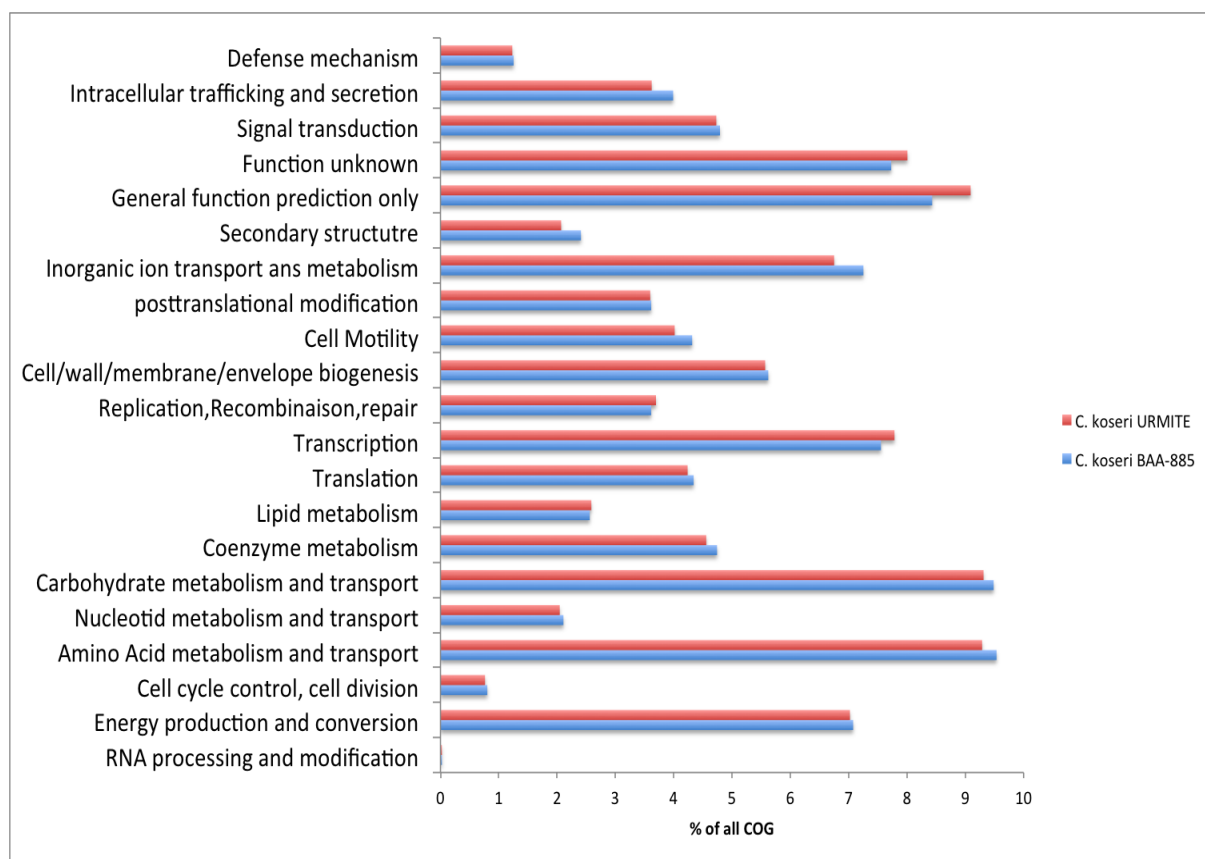

Supplement: Supplementary file 9 [file Image3.PDF]
