# Supplementary material for: Genomic Insights into a New Citrobacter koseri Strain Revealed Gene Exchanges with the Virulence-Associated Yersinia pestis pPCP1 Plasmid
Source: Front Microbiol. 2016 Mar 16;7:340. doi: 10.3389/fmicb.2016.00340 (PMC4793686; doi:10.3389/fmicb.2016.00340)
Supplement: Supplementary file 11 [file Image5.PDF]

Supplementary Figure S5: The Yersiniabactin locus

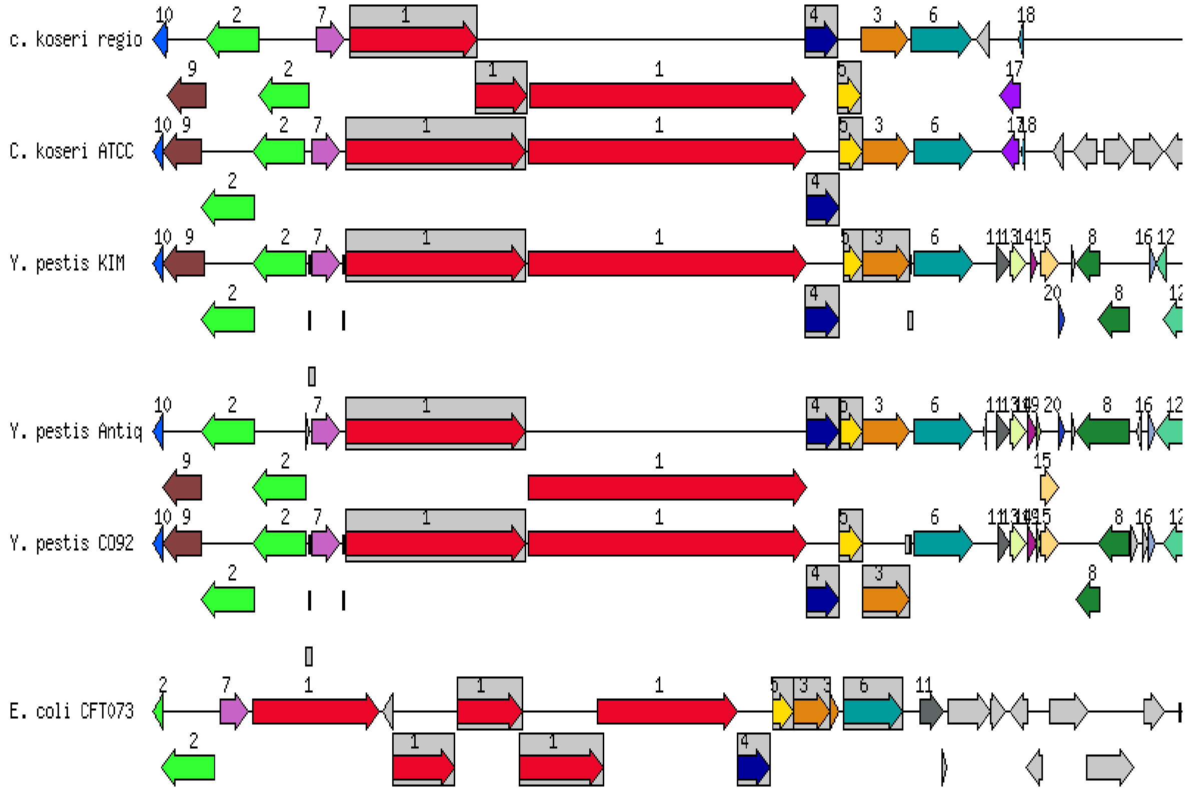

RAST region comparison focused on the Yersiniabactin locus of CKU (Top of the figure, named as C. koseri regio). Sets of genes with similar sequences are grouped with the same number and same color. The four most similar regions to those of the Yersiniabactin locus of our CKU belong to *C. koseri* ATCC BAA-895, *Yersinia pestis* KIM, Antiqua and CO92. Potential split gene for the gene number 1 in *C. koseri* URMITE compared with that of *C. koseri* BAA-895 and *Y. pestis* spp..
