# Supplementary material for: Genomic Insights into a New Citrobacter koseri Strain Revealed Gene Exchanges with the Virulence-Associated Yersinia pestis pPCP1 Plasmid
Source: Front Microbiol. 2016 Mar 16;7:340. doi: 10.3389/fmicb.2016.00340 (PMC4793686; doi:10.3389/fmicb.2016.00340)
Supplement: Supplementary file 12 [file Image6.PDF]

Supplementary Figure S6: Sequence comparison of the CKU tra operon

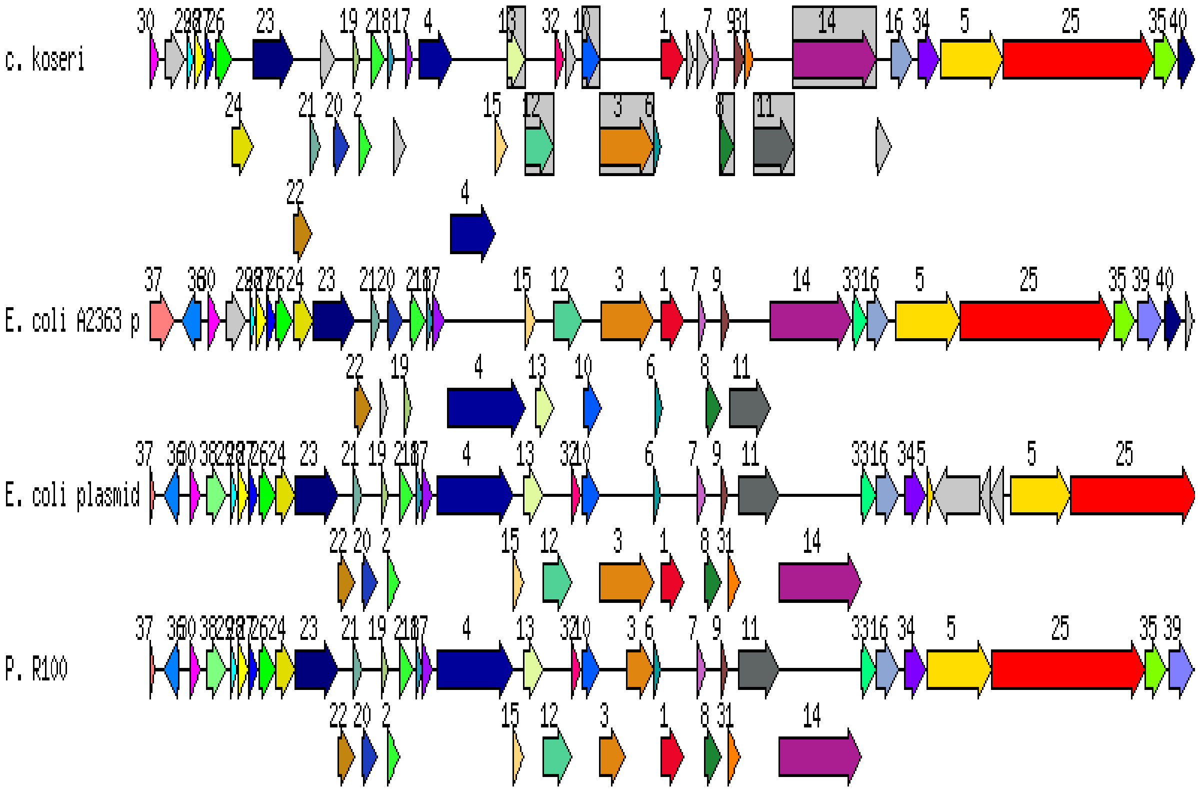

RAST region comparison focused on the tra operon of *Citrobacter koseri* URMITE. Sets of genes with similar sequences are grouped with the same number and same color. The four most similar regions to those of the tra operon of our *C. koseri* URMITE belong to *Escherichia* plasmids (*E. coli* A2363 pAPEC-O2-R, *E. coli* pO26I, *E.coli* pO42, *E.coli* pR100).
