# Supplementary material for: Genomic Insights into a New Citrobacter koseri Strain Revealed Gene Exchanges with the Virulence-Associated Yersinia pestis pPCP1 Plasmid
Source: Front Microbiol. 2016 Mar 16;7:340. doi: 10.3389/fmicb.2016.00340 (PMC4793686; doi:10.3389/fmicb.2016.00340)
Supplement: Supplementary file 13 [file Image7.PDF]

Figure S7: SNP-based phylogenetic tree of pCitro2

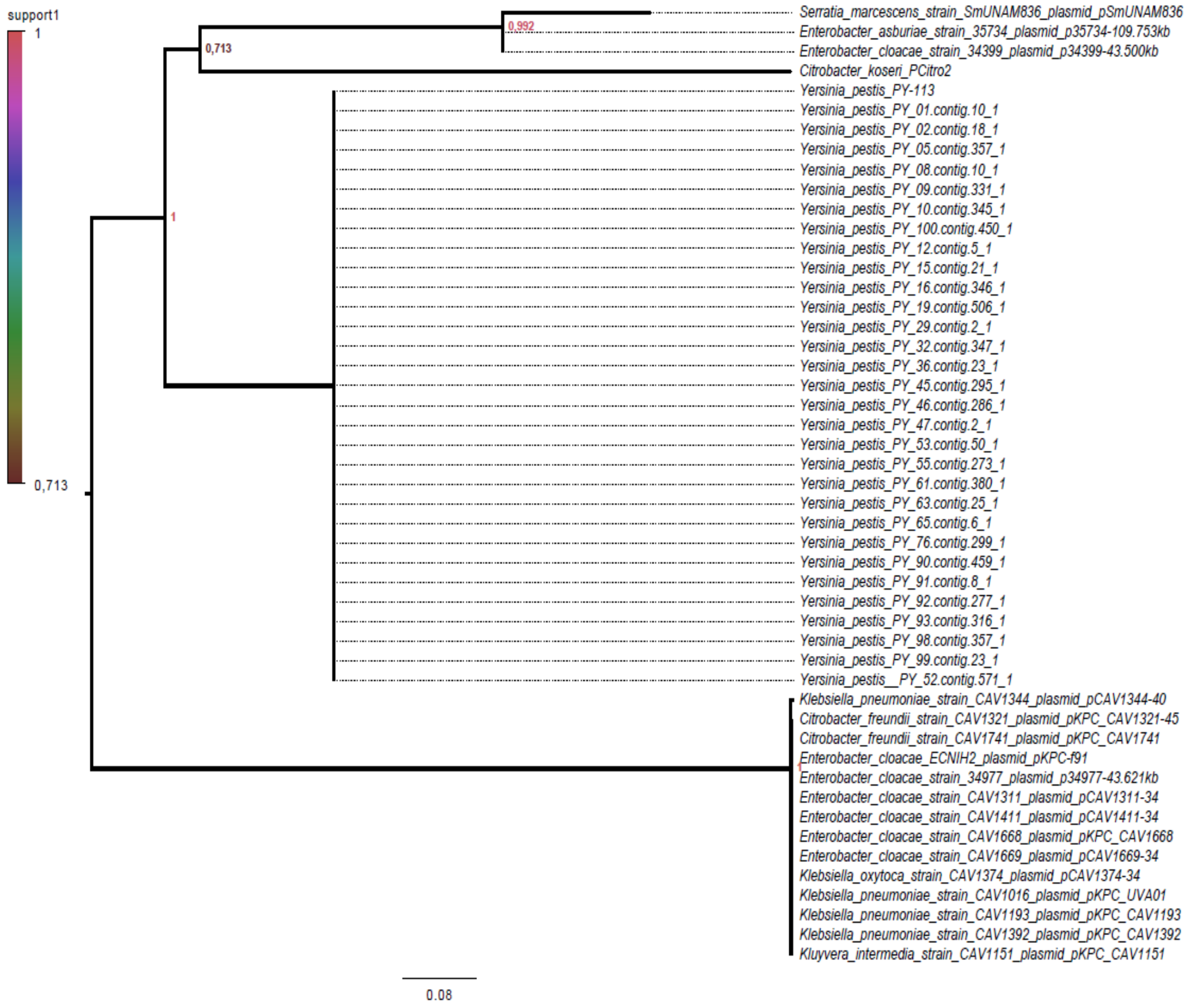

SNP-based phylogeny tree using genomic sequences retrieved from the best blast hits with the pCitro2 plasmid. The SNPs are identified in the core region of all the sequences. The branch supports are indicated as posterior probabilities.
