# Supplementary material for: Genomic Insights into a New Citrobacter koseri Strain Revealed Gene Exchanges with the Virulence-Associated Yersinia pestis pPCP1 Plasmid
Source: Front Microbiol. 2016 Mar 16;7:340. doi: 10.3389/fmicb.2016.00340 (PMC4793686; doi:10.3389/fmicb.2016.00340)
Supplement: Supplementary file 14 [file Image8.PDF]

Figure S8: Localization of non-synonymous mutations of the predicted Pla of pCitro1 using the Pla structure of *Y. pestis*

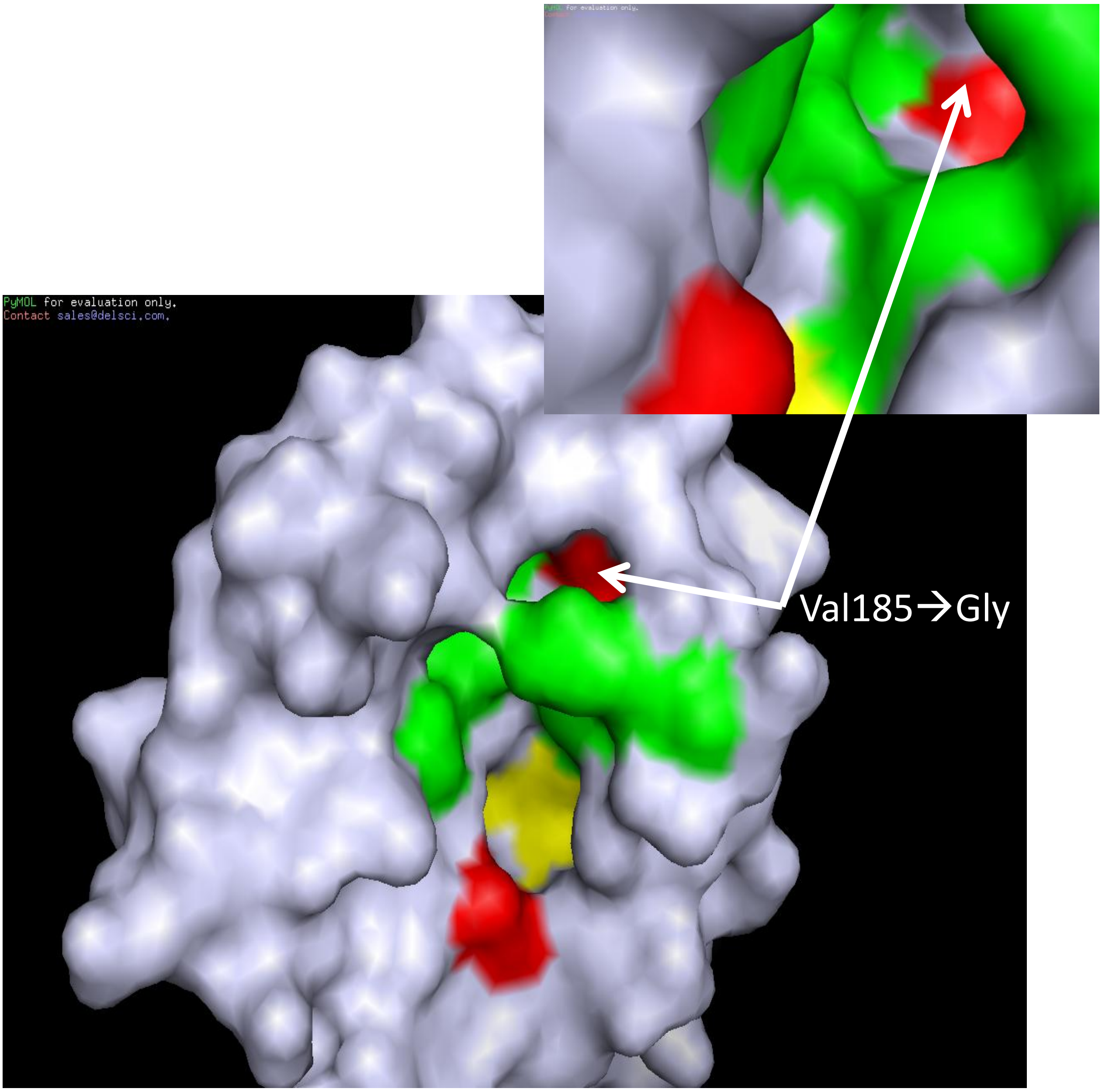

Green residues define the active site of Pla, S99, H101, D84, D86, D206, H208. The couple D206/H208 is crucial for the catalytic activity. In yellow, the hydrophobic pocket formed by residues E29, D204, E217. V185 is localized in the pocket near to the Pla active residues.
