# Supplementary material for: Genomic Insights into a New Citrobacter koseri Strain Revealed Gene Exchanges with the Virulence-Associated Yersinia pestis pPCP1 Plasmid
Source: Front Microbiol. 2016 Mar 16;7:340. doi: 10.3389/fmicb.2016.00340 (PMC4793686; doi:10.3389/fmicb.2016.00340)
Supplement: Supplementary file 15 [file Image9.PDF]

Figure S9: Phylogenetic analysis of the *Y. pestis* antivirulence lpxL

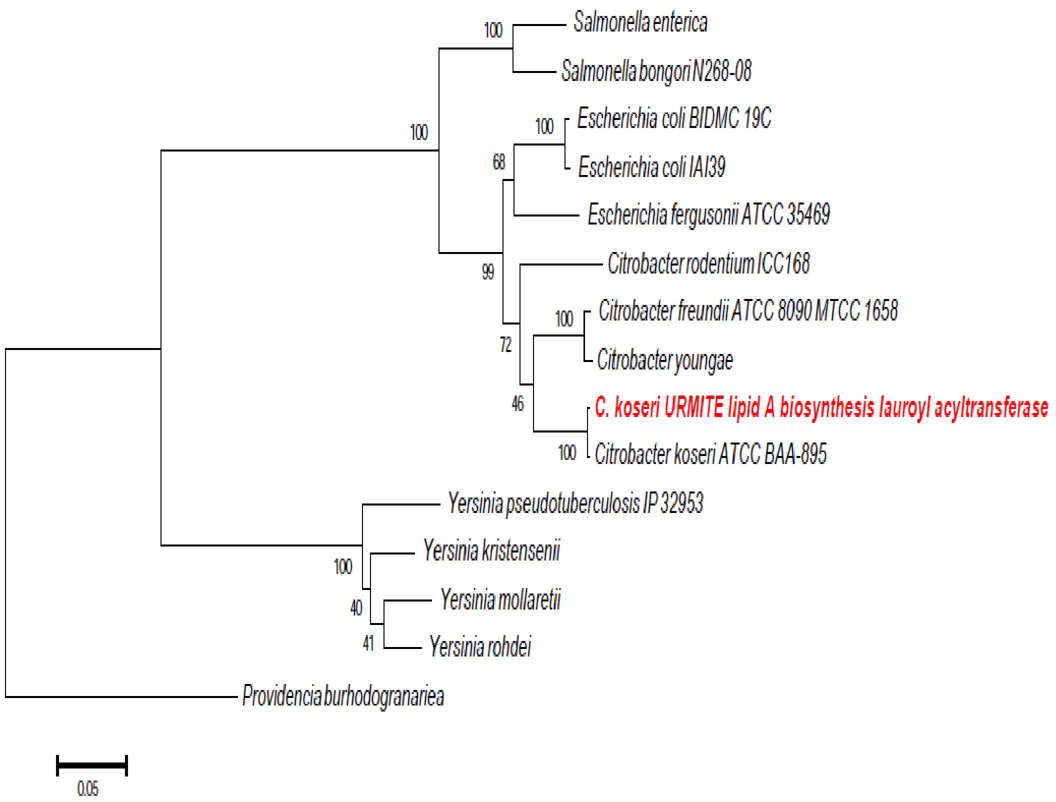

The antivirulence lpxL gene, a lauroyl acyltransferase, is absent in all the *Y. pestis* species but present in the *Y. pseudotuberculosis* ancestor. The lost of the antivilulence lpxL gene by *Y. pestis* has increased is pathogenicity. Homolog to the lpxL gene is found in Escherichia spp. Salmonella spp. and in Citrobacter spp. including *C. koseri* URMITE. The lpxL gene of Citrobacter is closely related to Escherichia spp. rather than Yersinia.
